# CpxR promotes the carbapenem antibiotic resistance of *Klebsiella pneumoniae* by directly regulating the expression and the dissemination of *bla*_KPC_ on the IncFII conjugative plasmid

**DOI:** 10.1080/22221751.2023.2256427

**Published:** 2023-09-06

**Authors:** Zhiyuan Liu, Jiahao Guan, Zhaoyan Chen, Cui Tai, Zixin Deng, Yanjie Chao, Hong-Yu Ou

**Affiliations:** a State Key Laboratory of Microbial Metabolism, Joint International Laboratory on Metabolic & Developmental Sciences, School of Life Sciences & Biotechnology, Shanghai Jiao Tong University, Shanghai, People’s Republic of China; b The Center for Microbes, Development and Health (CMDH), CAS Key Laboratory of Molecular Virology and Immunology, Shanghai Institute of Immunity and Infection, Chinese Academy of Sciences, Shanghai, People’s Republic of China; c Intensive Care Unit, First Affiliated Hospital of Guangxi Medical University, Nanning, People’s Republic of China

**Keywords:** Two-component system, CpxR, carbapenem resistance gene, plasmid conjugation, *Klebsiella pneumoniae*

## Abstract

*Klebsiella pneumoniae* is an important human pathogen known for its resistance to carbapenem antibiotics, especially the increasing carbapenem-resistant hypervirulent variants. The carbapenem resistance is mainly caused by the carbapenemase gene *bla*_KPC_ which was commonly found on the IncFII transferable plasmids in *K. pneumoniae* ST11 isolates in regions of China. However, the mechanisms of the plasmid-carrying *bla*_KPC_ regulation by the host strain are not clear. To investigate the chromosome-encoded two-component system (TCS) that regulates the carbapenem resistance of *K. pneumoniae* caused by *bla*_KPC_, twenty-four TCSs of a carbapenem-resistant classical *K. pneumoniae* ST11 clinical isolate were knocked out. The deletion mutation of the TCS regulator *cpxR* exhibited increased sensitivity to carbapenem, which could be restored by complementation with *cpxR in trans*. Electrophoretic mobility shift, isothermal titration calorimetry and DNase I footprinting results revealed that CpxR directly bound to the promoter DNA of *bla*_KPC_ and the binding was abolished by disrupting the DNA-binding domain in CpxR. The subsequent *in vivo* assays using the *lacZ* reporter system and qPCR showed that CpxR upregulates the transcription of *bla*_KPC_. Notably, CpxR was also found to activate the transfer of the *bla*_KPC_-carrying IncFII plasmid between the hypervirulent *K. pneumoniae* and *E. coli* isolates, in which CpxR promoted the transcription of the *tra* operon via binding to its promoter region. These results provide an important insight into the regulation of the host factor CpxR in the plasmid-carrying carbapenemase gene in the classical and hypervirulent *K. pneumoniae*.

## GRAPHICAL ABSTRACT

**Figure:**
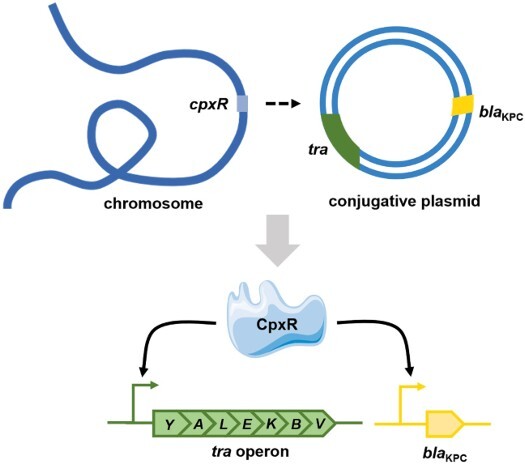

## Highlights

- CpxR contributes to the carbapenem antibiotics resistance of classical and hypervirulent *K. pneumoniae*.
- CpxR binds to the promoter region of *bla*_KPC_ and upregulates the *bla*_KPC_ transcription.
- CpxR binds to the promoter of the *tra* operon and activates the conjugation of the carbapenem-resistant plasmid.

## Introduction

*Klebsiella pneumoniae* is one of the most prominent carbapenem-resistant Enterobacteriaceae (CRE). The carbapenem resistance rate of classical *K. pneumoniae* (cKP) increased over 8-fold from 2005 to 2018 in China [1]. Notably, the hypervirulent *K. pneumoniae* (hvKP) used to be sensitive to antimicrobial agents but now variants of hvKP with high degrees of carbapenem resistance have increasingly emerged [2]. The carbapenem resistance of *K. pneumoniae* is mainly caused by the carbapenemase gene *bla*_KPC_ [3], which is much more frequently detected than other carbapenemase genes such as *bla*_OXA-48_, *bla*_NDM-1_ and *bla*_IMP-1_ [4, 5]. *K. pneumoniae* has the capability of acquiring new plasmids that code for antibiotic resistance determinants and/or virulence factors [6]. In the sequence type (ST) 11 carbapenem-resistant *K. pneumoniae*, the most prevalent *K. pneumoniae* strains in China, the *bla*_KPC_ genes were commonly found in the IncFII transferable plasmids [7]. Since the acquisition of these resistant plasmids readily turns host bacteria resistant to carbapenems, *bla*_KPC_ has been considered an autonomous gene that may be constitutively expressed [8]. However, little was known whether there exists any interaction of the *bla*_KPC_ gene in the transferable plasmid with the regulation factors encoded by the host bacterial chromosome.

Bacterial two-component systems (TCSs) are important signal transduction systems that sense and respond to changes in the external environment. TCS usually consists of two components: a cell membrane protein histidine kinase (HK) and a cytosolic protein response regulator (RR). In response to extracellular changes, HK autophosphorylates at the conserved histidine residues and transfers the phosphoryl group to cognate RR. The phosphorylated RR binds to promoters and controls gene expression at the transcriptional level [9]. CpxAR is a TCS monitoring the inner-membrane homeostasis, with CpxA as HK and CpxR as RR [10]. Upon activation, CpxR strongly induces the transcription of downstream gene *cpxP*, whose mRNA is further processed into a small regulatory RNA CpxQ [11]. Notably, the CpxAR system has been reported to play a crucial role in antimicrobial resistance in a variety of bacterial pathogens [12–16]. Constitutive activation of CpxR in *E. coli* and *Salmonella* leads to intrinsic resistance to some but not all bactericidal drugs including aminoglycoside, hydroxyurea and β-lactam [17–19], despite the mechanisms remaining little understood. The inactivation of *cpxAR* in *K. pneumoniae* rendered bacteria more sensitive to cefepime and chloramphenicol [13]. The CpxAR system seems to regulate intrinsic resistance pathways by impacting drug transport and membrane porins [20–23]. However, it was unclear whether the chromosome-borne CpxAR directly regulates drug-resistance genes carried on the foreign, mobile plasmids.

We previously reported the carbapenem-resistant ST11 cKP clinical isolate HS11286, in which *bla*_KPC_ was encoded on an IncFII-IncR conjugative plasmid called pKPHS2 [24]. The deletion of pKPHS2-borne *bla*_KPC_ significantly decreased the level of meropenem resistance exhibited by the resultant mutant strains [25]. Here, we investigated the expression, regulation and interaction between the chromosome-coding TCS and the self-transferable plasmid-carrying *bla*_KPC_ gene of *K. pneumoniae*.

## Materials and methods

### Strains, plasmids and primers

All strains and plasmids used in this study are listed in Table S1. All primers used in this study are listed in Table S2. All deletion mutants were generated by lambda red recombination and allelic exchange using the suicide vector pKOBEG-Apra as described in Bi *et al.* [25]. Polymerase chain reaction (PCR) was conducted using specific forward primer and reverse primer designed within the gene deletion region. If the DNA product was obtained from the wild-type but not from the mutant, it was considered successful construction of the mutant. The low-copy vector pXG10 with respective genes was used for complementation. All *E. coli* and *K. pneumoniae* strains were cultured in LB broth or on LB agar with appropriate antibiotics.

### Antimicrobial susceptibility testing

The disc diffusion assay was performed according to the Clinical and Laboratory Standards Institute standard (CLSI, 2023). Fresh bacterial cells were made into bacterial suspensions with a McFarland turbidity of 0.5. After evenly spreading on Mueller-Hinton agar, a Meropenem Antimicrobial Susceptibility disc (Oxoid, 10 μg) was placed in the centre of the plate. After incubating at 37°C for 16 h, the diameter of the zone of inhibition was measured.

For the agar screening, a spot assay was used to determine bacterial sensitivity to meropenem. In brief, the bacterial suspensions with an OD_600 nm_ value of 0.5 were diluted by a 10-fold gradient. Then 5 μl of all suspensions were carried out on LB agar with or without meropenem respectively. The concentration of meropenem used for *K. pneumoniae* HS11286 and its mutants was 2 μg/ml. And 1 μg/ml was used for *K. pneumoniae* 293Z and its mutants, 0.25 μg/ml was used for *E. coli* C600 and its mutants.

The minimal inhibitory concentrations (MICs) were determined using the broth microdilution method according to the CLSI standard (2023). The tested strains were inoculated at a final concentration of 0.5 McFarland standard into CAMHB (Cation-Adjusted Mueller-Hinton Broth) medium supplemented with meropenem at concentrations of 64, 32, 16, 8, 4, 2, 1, 0.5, 0.25, 0.125, 0.06, and 0.03 μg/ml. When determining the MIC of Meropenem-vaborbactam (MEV), a final concentration of 8 µg/ml vaborbactam was added to the mixture of the aforementioned bacterial cultures and meropenem. The cultures were incubated at 37°C under ambient air for 16 h, and bacterial growth was observed to determine the MIC. *E. coli* ATCC 29522 was used as the quality control strain.

### Expression and purification of proteins

The wild-type *cpxR* gene and its mutation variants genes were cloned into vector pET28a(+) using primers listed in Table S2, and transferred into *E. coli* BL21(DE3). Cells containing plasmids were grown at 37°C with shaking. When an OD_600 nm_ of 0.5 was reached, protein expression was induced overnight at 16°C with 0.2 mM Isopropyl β-D-1-thiogalactopyranoside (IPTG). Subsequently, proteins were purified from cell lysis supernatant with cobalt metal affinity resin (Takara). Contaminating nucleic acid was removed by HiTrap Heparin HP (GE Healthcare) or HiTrap Capto Q (GE Healthcare).

### Electrophoretic mobility shift assays

Electrophoretic mobility shift assays (EMSAs) were carried out by mixing wild-type or mutated CpxR at a range of concentrations with EMSA/Gel-Shift Binding Buffer (poly(dI-dC), DTT, Glycerol, EDTA, NaCl, MgCl_2_, Tris) (Beyotime) at room temperature for 10 min. Then, the 2 pmol promoter DNA probes with a 6-FAM modification at the 5-end were added and incubated for 20 min. The mixtures were then subjected to electrophoresis in native 6% polyacrylamide gel at 100 V for 60 min.

### DNase I footprinting assay

DNase I footprinting assays were performed according to the method described by Wang *et al.* [26]. Four hundred ng promoter DNA probes with a 6-FAM modification at the 5-end were incubated with or without CpxR in a total volume of 40 µl in binding buffer (50 mM Tris-HCl pH 8.0, 100 mM KCl, 2.5 mM MgCl_2_, 0.2. mM DTT, 2 μg salmon sperm DNA and 10% glycerol) for 30 min at 25°C. Then 10 µl solution containing about 0.015 unit DNase I (Promega) and 100 nmol freshly prepared CaCl_2_ was added and further incubation was performed at 37°C for 1 min. The reaction was stopped by adding 140 µl stop solution (200 mM unbuffered sodium acetate, 30 mM EDTA and 0.15% SDS). Samples were extracted with phenol/chloroform and precipitated with ethanol. Pellets were dissolved in 30 µl MiliQ water. The preparation of the DNA ladder, electrophoresis and data analysis were the same as described before, except that the GeneScan-LIZ600 size standard (Applied Biosystems) was used.

### Isothermal titration calorimetry

Isothermal titration calorimetry experiments were performed on a TA NANO system. Genomic DNA was extracted from *K. pneumoniae* HS11286 and used as a template for PCR amplification of the promoter DNA fragment of *bla*_KPC_, using Pkpc403-F/R primers (listed in Table S2). The purified CpxR protein (0.1 μM) and the promoter DNA of *bla*_KPC_ (0.01 μM) were dissolved in HEPES (4-(2-hydroxyethyl)−1-piperazineethanesulfonic acid) (pH 7.4). Sixty microliters purified CpxR protein was injected into the sample cell filled with 300 μl promoter DNA of *bla*_KPC_, and the injection was repeated 20 times with an equilibrium interval of 200 s. The experiment was conducted at 25°C. The equilibrium dissociation constant *K_D_* was obtained using the nanoAnalyzer software. 0.4 μM the CpxR-R195H protein and 0.04 μM promoter DNA of *bla*_KPC_ were used.

### Real-time quantitative PCR (qRT-PCR)

Total RNA was isolated using the RNeasy Mini Kit (Qiagen). Then gDNA was removed and cDNA was produced by PrimeScript^TM^ RT reagent Kit with gDNA Eraser (Takara). Finally, qPCR was performed using Hieff UNICON® qPCR SYBR Green Master Mix (YEASEN) and *gapA* was used as an internal control. The gene expression level was calculated by the 2^-ΔΔCT^ method.

### β-galactosidase activity assay

The β-galactosidase activity was quantified based on the hydrolysis of O-Nitrobenzene-β-D-galactopyranoside (ONPG). Bacteria were subcultured in LB broth to logarithmic phase (OD_600 nm_ of 0.4 ∼ 1.0), then cells were pelleted and resuspended in an equal amount of Z buffer (0.06 M Na_2_HPO_4_, 0.04 M NaH_2_PO_4_, 0.01 M KCl, 0.001 M MgSO_4_, 0.05 M β-mercaptoethanol). One millilitre of cells (OD_600 nm_ determined after blank against the Z buffer) were permeabilized by adding 100 μl chloroform and 50 μl 0.1% SDS and was equilibrated for 5 min in a 28°C water bath. The reaction was started by adding 0.2 ml O-nitropheny1-β-D-galactoside (ONPG, 4 mg/ml) at 28°C and was stopped after the yellow colour has developed by adding 0.5 ml 1 M Na_2_CO_3_. The time of reaction (T) was recorded precisely with a timer. Then the mixture was spined 5 min at maximum to remove debris and chloroform. OD_420 nm_ and OD_550 nm_ of supernatant were measured. Units of activity were calculated with the formula as follows. 1)

### Conjugation assay

Bacteria were subcultured in LB broth to the logarithmic phase. Two millilitres of donor cells and recipient cells were washed with 10 mM MgSO_4_ for 3 times, resuspended in 40 μl of 10 mM MgSO_4_, mixed and then 20 μl was inoculated on LB agar for 12 h at 37°C. The mixtures of donors, recipients and transconjugants cells were resuspended in Phosphate Buffered Saline (PBS) and serially diluted. The serial dilutions were plated on LB agar with appropriate antibiotic (listed in Table S3) and the CFU of the transconjugants and donors cells were counted. The transconjugants were further validated by PCR. The gene *bla*_KPC_ was located on pKPHS2 and used as a marker for the presence of plasmid. The gene *khe* exists only in *K. pneumoniae* but not in *E. coli* [27], which can distinguish between donors and recipients. The conjugation frequency was calculated as the ratio of transconjugants to donors.

### Statistical analysis

The data of promoter activity and qRT-PCR were derived from three independent assays. Student’s *t*-test (unpaired, equal variance, two-sided) was performed using R package (https://www.r-project.org/). Statistical significance was considered when *p *≤ 0.05. * indicates *p* < 0.05; ** indicates *p* < 0.01; *** indicates *p* < 0.001.

## Results

### CpxR contributes to the resistance of *K. pneumoniae* to carbapenem antibiotics

**Figure 1.**
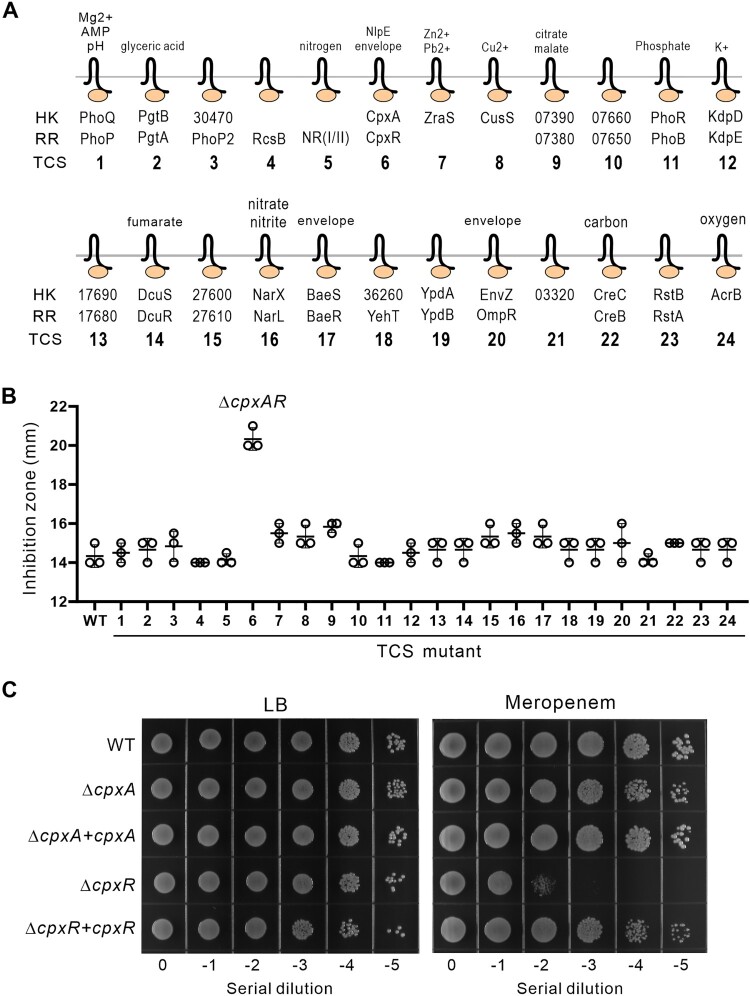
Identification of a two-component system that is required for resistance to β-lactam antibiotics in *K. pneumoniae*. **(A)** Twenty-four TCSs were individually deleted on the chromosome of *K. pneumoniae* HS11286. Abbreviations: HK, histidine kinase; RR, response regulator; TCS, two-component system; AMP, antimicrobial peptides. **(B)** Diameter of inhibition zone of 24 TCS deletion mutants to meropenem. **(C)** Growth of bacterial serial dilutions in the presence or absence of meropenem.

To better understand the involvement of TCS in antibiotic resistance, we first predicted all the potential TCSs encoded in the chromosome of ST11 cKP HS11286 using the Prokaryotic 2-Component Systems database (P2CS) [28]. Of all the TCSs that have been predicted and annotated (Table S4), we have successfully constructed mutants for 24 TCSs, each harbouring a chromosomal deletion of a TCS (Figure 1A, Figure S1). Using disk diffusion test, we discovered that the ΔTCS6 strain (*K. pneumoniae* HS11286 harbouring a deletion of *cpxAR*) showed an increased sensitivity to meropenem (a kind of carbapenem antibiotics) compared to the wild type (Figure 1B). According to the CLSI 2023, the ΔTCS6 strain exhibited intermediate resistance to meropenem, while the wild-type and other mutants were resistant. Further dissecting the responsible gene in the *cpxAR* bicistronic operon (Figure S2) confirmed the direct involvement of the *cpxR* gene, which encodes the response transcriptional regulator CpxR. A clean mutant of *cpxR* (Δ*cpxR*) showed impaired resistance to meropenem, which could be fully restored by complementation with a wild-type *cpxR* gene *in trans* (Figure 1C, Table S5).

### CpxR directly binds to the promoter region of *bla*_KPC_

**Figure 2.**
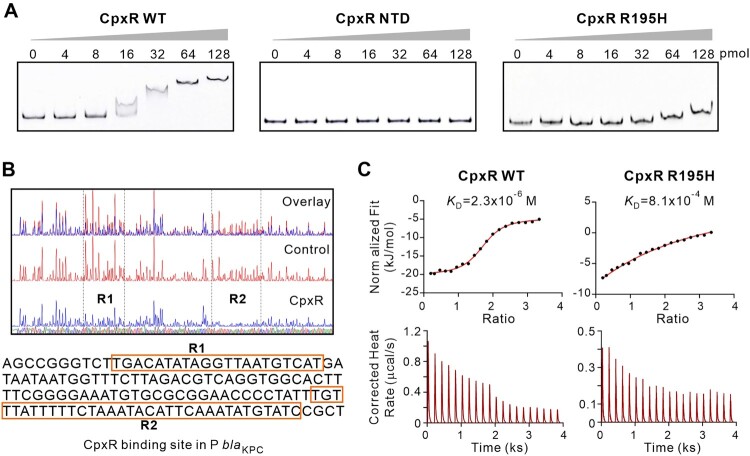
CpxR recognizes and binds to the promoter region of *bla*_KPC_. **(A)** Electrophoretic mobility shift assay (EMSA) using increasing amounts of wild-type CpxR, CpxR^NTD^ (CpxR missing C-terminal domain), and CpxR^R195H^ with 2 pmol of FAM-labeled promoter of *bla*_KPC_. (Negative Controls, Figure S8) **(B)** DNase I footprinting assay. The FAM-labeled promoter region of *bla*_KPC_ was incubated with or without CpxR protein, then digested by DNase I. Electropherograms indicated the sequence of protection region. The peaks at R1 and R2 were significantly decreased in the presence of CpxR, indicating direct binding sites of CpxR. **(C)** Isothermal titration calorimetry assay of the *bla*_KPC_ promoter region with CpxR and CpxR^R195H^. The genetic organizations of CpxR, CpxR^NTD^, and CpxR^R195H^ were depicted in Figure S3.

Meropenem resistance in *K. pneumoniae* is mainly due to the expression of β-lactamase *K. pneumoniae* carbapenemase (KPC, encoded by *bla*_KPC_ gene), which efficiently hydrolyzes carbapenem-like drugs and mediates resistance to β-lactam antibiotics. Using vaborbactam [29], an inhibitor of β-lactamase KPC, carbapenem-resistant *K. pneumoniae* becomes susceptible to carbapenem (Table S5), supporting the alteration of carbapenemase as the cause for the change of meropenem resistances. We hypothesized that the impaired meropenem resistance in Δ*cpxR* may be due to transcriptional regulation of *bla*_KPC_ by CpxR, a member of the OmpR/PhoB transcriptional regulator family with a C-terminal DNA-binding domain (DBD) (Figure S3A). Using electrophoretic mobility shift assay (EMSA), we indeed found that CpxR directly binds to the promoter region of *bla*_KPC_ with high affinity (Figure 2A). As expected, this binding was reduced by disrupting the DNA-binding domain in CpxR (Figure 2A), either by removing the C-terminal domain (CTD) or by mutating the crucial Arg_195_ residue to a histidine [30]. The Isothermal titration calorimetry analysis, which measures the binding affinity between CpxR and the P*bla*_KPC_ sequence, showed that the R195H mutation significantly reduced the DNA binding affinity of the CpxR by ∼100 fold (Figure 2C). We have further determined the CpxR-binding sites using DNase I footprinting assay and revealed two regions in the *bla*_KPC_ promoter that were protected by CpxR (Figure 2B), *i.e.* 5’-TGACATATAGGTTAATGTCAT-3’ and 5’-TGTTTATTTTTCTAAATACATTCAAATATGTATC-3’. The sequence alignment of the obtained CpxR binding sites against the completely sequenced *K. pneumoniae* genomes available in the NCBI RefSeq database (Figure S4) revealed that the CpxR binding site sequences are present in 239 out of 416 (57%) of the promoter regions of *bla*_KPC_ genes (Figures S5 and S6). Notably, this binding was not only observed for the *bla*_KPC_ promoter region in *K. pneumoniae* Tn*1721* of ST11, the dominant KPC-producing cKP clone in China, but also observed by EMSA for the promoter from Tn*4401*b of ST258**,** the dominant carbapenem-resistant *K. pneumoniae* clone in Europe and the United States (Figure S7). However, the CpxR binding sites to the *bla*_KPC_ promoter region in Tn*4401*b were different from those of Tn*1721* and require further investigation (Figures S5 and S6).

### CpxR upregulates the transcription of *bla*_KPC_ and carbapenem resistance

**Figure 3.**
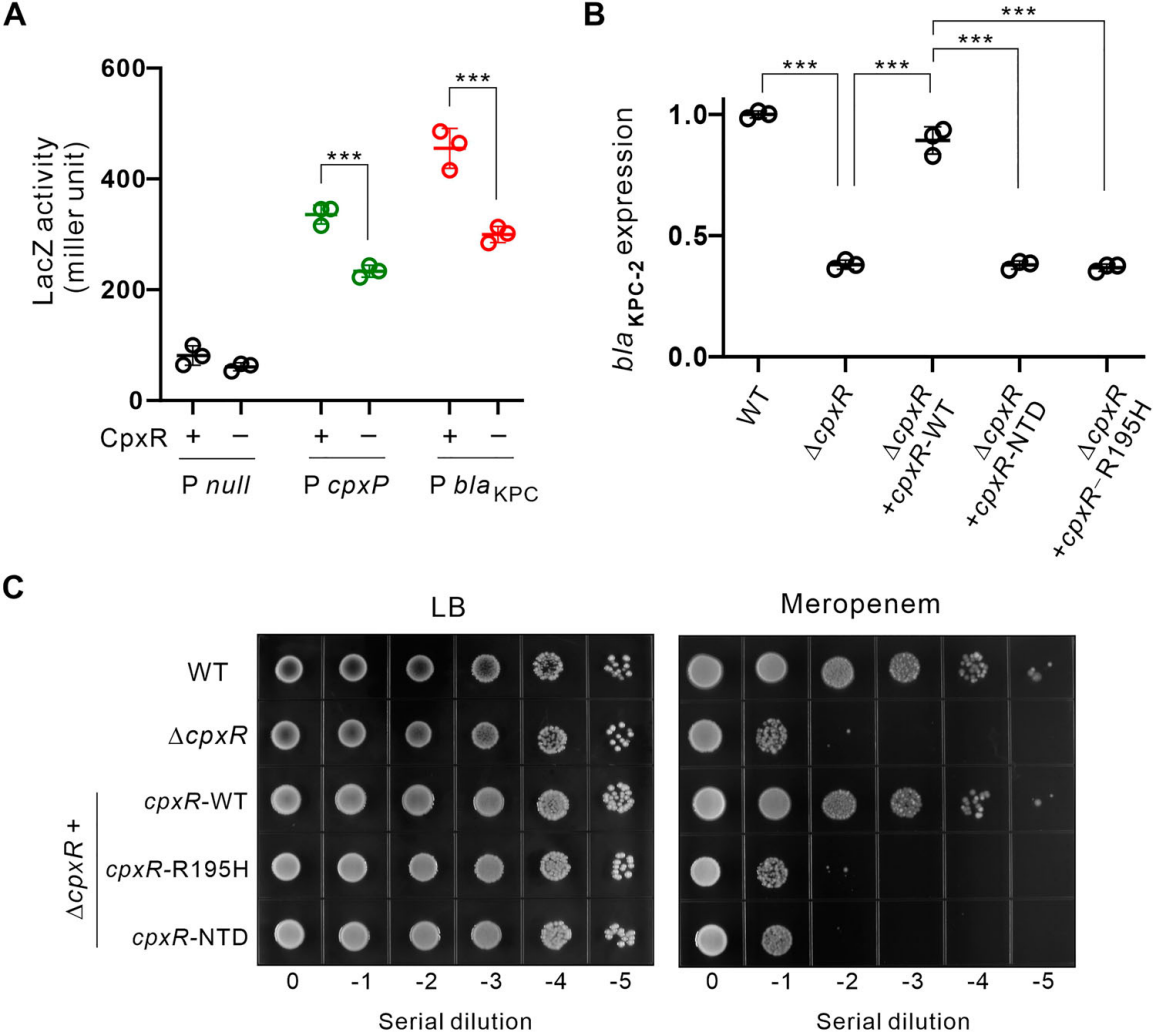
CpxR promotes the transcription of *bla*_KPC_. **(A)** The activity of *lacZ* reporter fused to respective promoter was determined in the presence or absence of *cpxR.* The promoter of *cpxP* was used as a positive control [11]. The null promoter was used as a negative control. This experiment was performed in a heterologous system using *E. coli* DH5ɑΔ*cpxR* as a surrogate host, in which the *K. pneumoniae cpxR* gene was provided on plasmid. **(B)** The expression level of *bla*_KPC_ in the wild-type *K. pneumoniae,* Δ*cpxR* mutant, complementation of Δ*cpxR* with a wild-type *cpxR*, complementation of Δ*cpxR* with *cpxR*^NTD^ (CpxR N-terminal domain), complementation of Δ*cpxR* with *cpxR*^R195H^. **(C)** Bacterial growth in the presence or absence of meropenem using 10-fold serial dilutions.

Having established a direct binding of CpxR to the *bla*_KPC_ promoter, we next thought to analyze the impact of CpxR on *bla*_KPC_ transcription using a *lacZ* transcriptional reporter system. The 403 bp region of the *bla*_KPC_ promoter was cloned and fused to a promoterless *lacZ* gene, and the β-galactosidase activity was examined. As shown in Figure 3A, the transcription of *bla*_KPC_-*lacZ* was significantly reduced when *cpxR* was absent. A similar reduction was also observed for the P*cpxP* promoter, a known CpxR-dependent promoter as a positive control [11]. Further analysis at the mRNA level showed consistent results as above (Figure 3B). The expression of *bla*_KPC_ mRNA was only about one-third of the wild-type (−2.631 fold, *p* < 0.001) when *cpxR* was deleted. And the expression of KPC was decreased by half when *cpxR* was deleted (Figure S9). This reduction was restored by a *trans*-complementation of the wild-type *cpxR* gene, but not by the CpxR mutants without DNA-binding capacity (NTD, R195H). As expected, these two CpxR mutants also failed to restore the carbapenem resistance phenotype, whereas the WT CpxR did (Figure 3C, Table S5). Together, these results confirm that CpxR upregulates the transcription of carbapenem-hydrolyzing class A β-lactamase KPC from the large conjugative plasmid pKPHS2 and promotes carbapenem resistance in cKP.

### CpxR activates the transcription of *tra* operon and plasmid conjugation

**Figure 4.**
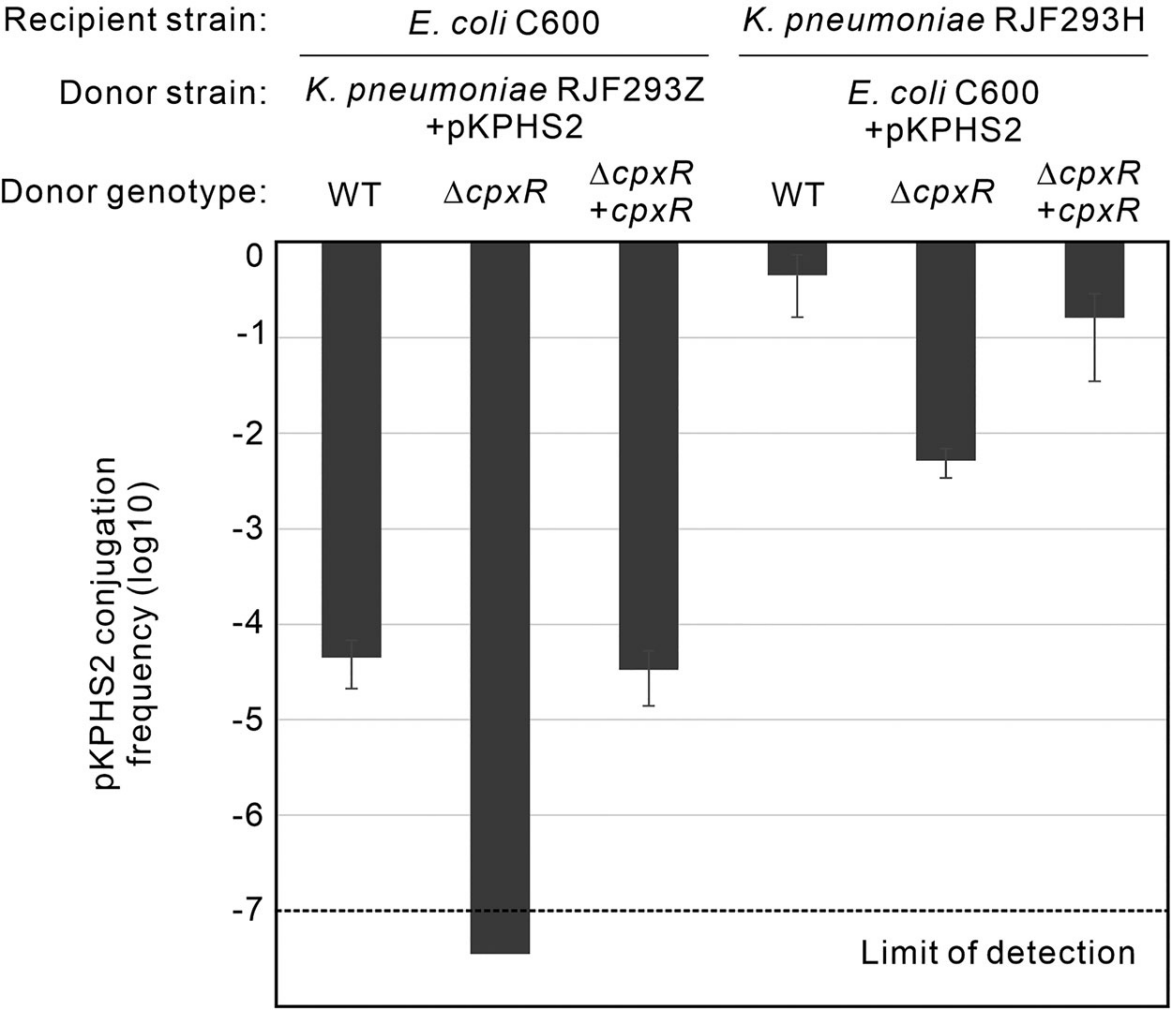
CpxR is required for the efficient transfer of the IncFII *bla*_KPC_-carrying pKPHS2 plasmid. Conjugation frequency of the pKPHS2 was determined using *K. pneumoniae* RJF293Z strains as donor and *E. coli* C600 as recipient; or using *E. coli* C600 strains as donor and RJF293H as recipient. (Verification of the pKPHS2 conjugation between *E. coli* C600 and *K. pneumoniae* RJF293, Figure S13)

Initially identified as a major regulator of the conjugative plasmid expression (cpx), CpxR has been established to inhibit the conjugation of F plasmid in *E. coli* [31, 32]. We wondered whether CpxR regulates the conjugation of the *bla*_KPC_-carrying IncFII self-transferable plasmid between the cKP, hvKP and *E. coli* isolates. First, we transferred pKPHS2, the *bla*_KPC_-carrying IncFII plasmid of cKP HS11286, to RJF293Z as the donor to test the conjugation frequency of pKPHS2 with *E. coli* C600 as the recipient (Figure S12A). Strikingly, the conjugation frequency of pKPHS2 was strongly reduced to below the limit of detection in the Δ*cpxR* donor strain of *K. pneumoniae* (Figure 4)*.* Then, in the second round of conjugation, *E. coli* C600 + pKPHS2 was employed as the donor, and the hvKP strain RJF293H was used as the recipient (Figure S12B). Again, we observed a strong reduction (∼100-fold) in conjugation efficiency with the Δ*cpxR* mutant compared to the WT donor (Figure 4), supporting that CpxR is necessary for the efficient transfer of the IncFII *bla*_KPC_-carrying pKPHS2 plasmid by conjugation.

**Figure 5.**
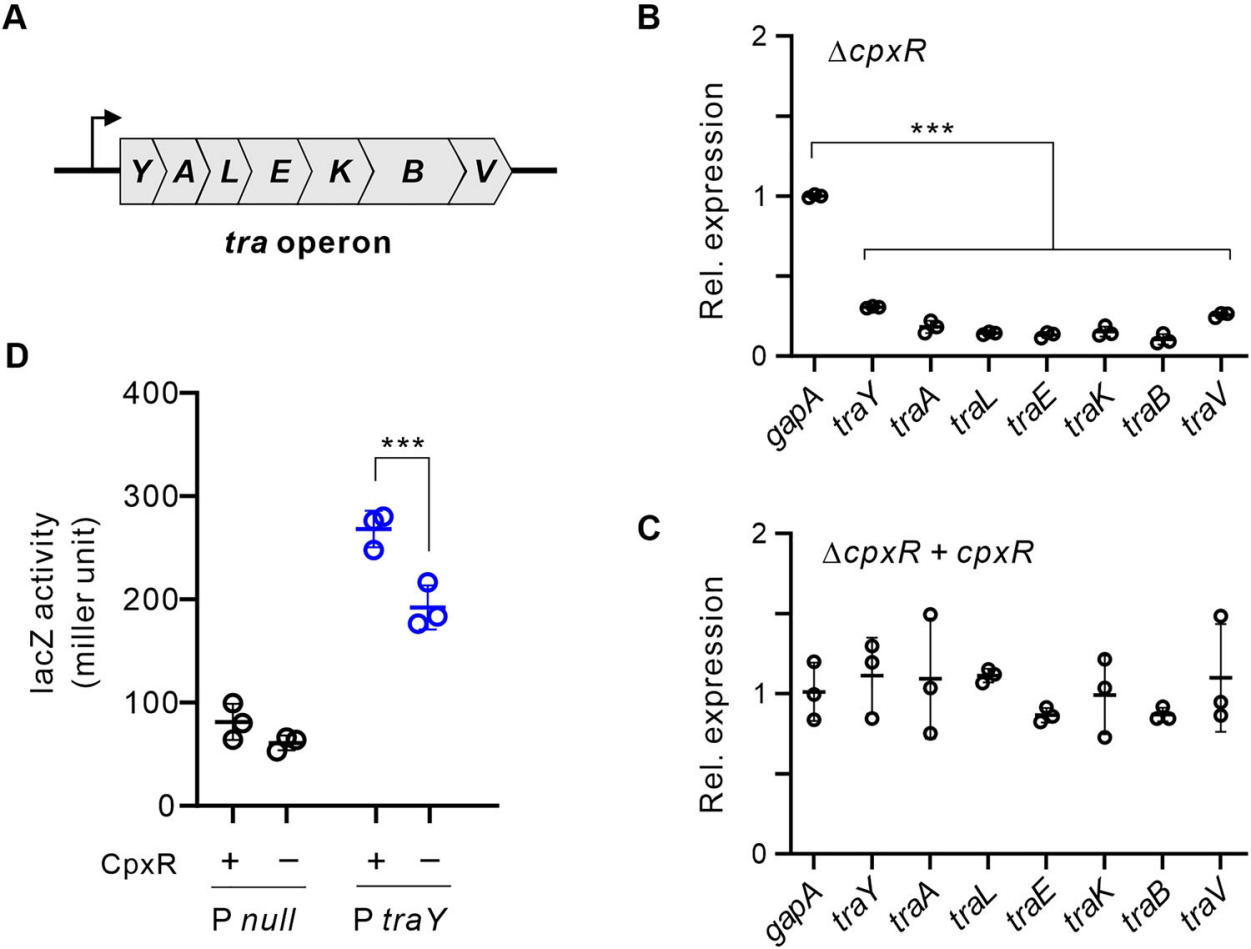
CpxR promotes the transcription of *tra* operon on the IncFII *bla*_KPC_-carrying plasmid pKPHS2. **(A)** Genetic organization of the *tra* operon. The genes *traY*, *traA*, *traL*, *traE*, *traK, traB* and *traV* form a polycistron that is transcribed from a promoter upstream (bended arrow). **(B)** The expression of *tra* genes in Δ*cpxR* mutant, using *gapA* as a control. **(C)** The expression level of *tra* genes in Δ*cpxR* complemented with a wild-type *cpxR*. **(D)** The beta-galactosidase activity of *lacZ* fused to the *traY* promoter was determined in the presence or absence of *cpxR*. The null promoter was used as a negative control. This experiment was performed in a heterologous system using *E. coli* DH5ɑΔ*cpxR* as a surrogate host, in which the *K. pneumoniae cpxR* gene was provided on plasmid.

**Figure 6.**
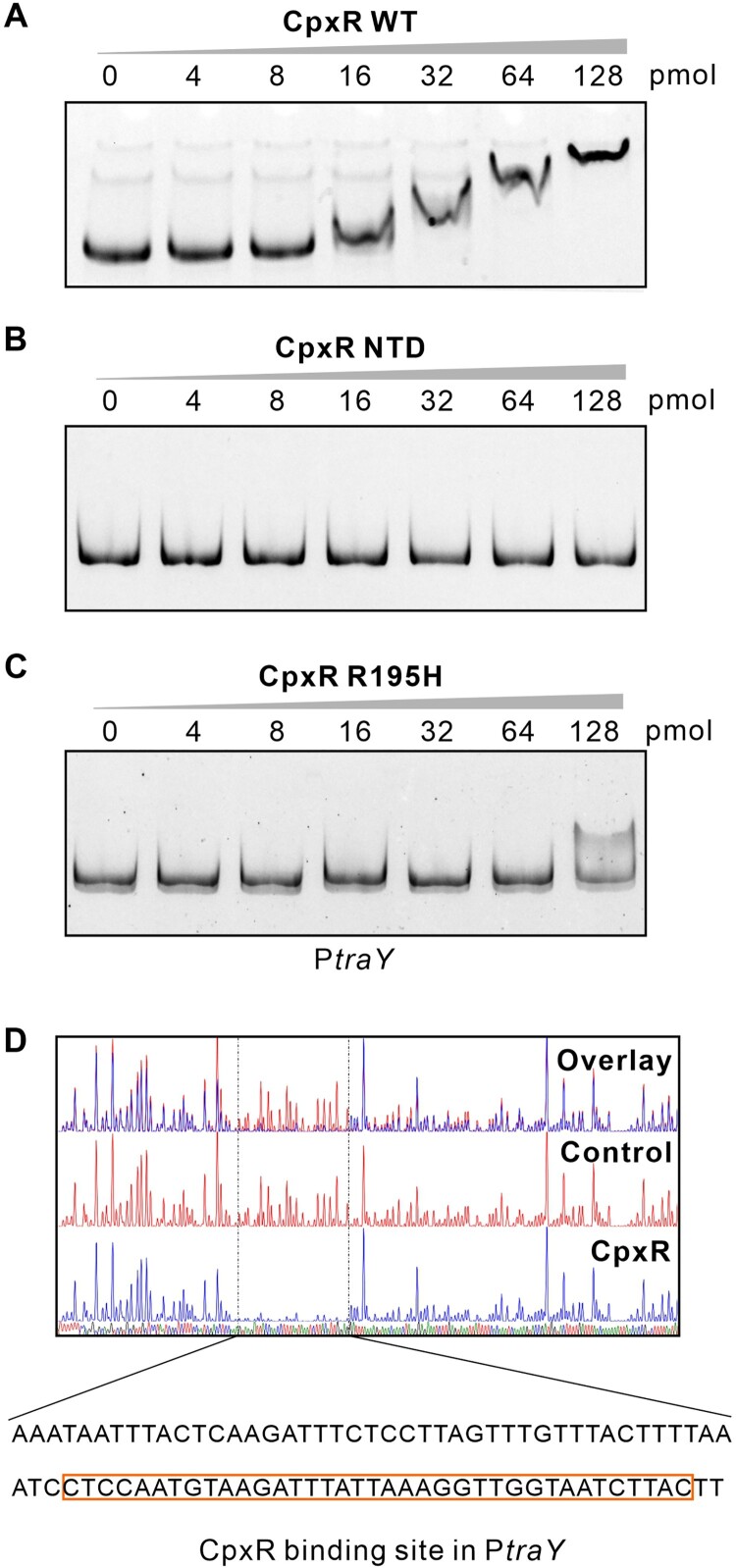
CpxR binds to the *traY* promoter. **(A-C)** EMSA using 2 pmol FAM-labeled *traY* promoter with increasing amount of wild-type CpxR **(A),** CpxR^NTD^ (CpxR missing C-terminal domain) **(B)**, or CpxR^R195H^ **(C)** (Negative Control, Figure S11). **(D)** DNase I footprinting assay. The FAM-labeled *traY* promoter was incubated with or without CpxR protein, then digested by DNase I. Electropherograms indicated the CpxR protection region. The peaks in the dashed box were significantly decreased, indicating the binding site of CpxR. The organizations of CpxR, CpxR^NTD^, CpxR^R195H^ were depicted in Figure S3.

The pKPHS2 plasmid of *K. pneumoniae* HS11286 encodes the *tra* operon required for conjugation (Figure 5A). This operon expresses a long polycistronic mRNA starting with *traY* as the first gene (Figure S10). To understand how CpxR regulates the conjugation of pKPHS2, we analyzed the expression of *tra* genes using qRT-PCR. The results showed that the expression of all seven *tra* genes was significantly down-regulated when *cpxR* was deleted (Figure 5B), and was again restored to the WT level in the CpxR complementation strain (Figure 5C). Using the *traY-lacZ* transcriptional reporter assay, we have confirmed that CpxR acts at the transcriptional level to promote the expression of *tra* operon (Figure 5D). To further establish a direct binding of CpxR to the *traY* promoter, we performed EMSA assay with the *traY* promoter DNA fragment. The results showed that the WT CpxR protein directly binds to the *traY* promoter (Figure 6A), whereas the CpxR mutants did now show any productive interaction (Figure 6BC). The CpxR binding site in the *traY* promoter has been determined using a DNase I protection assay (Figure 6D), *i.e*. 5’-CTCCAATGTAAGATTTATTAAAGGTTGGTAATCTTAC-3’. Altogether, our results showed that CpxR promotes conjugation by transcriptional activation of the *tra* operon on pKPHS2 in cKP.

### CpxR is the host factor promoting carbapenem resistance in the recipient strain

**Figure 7.**
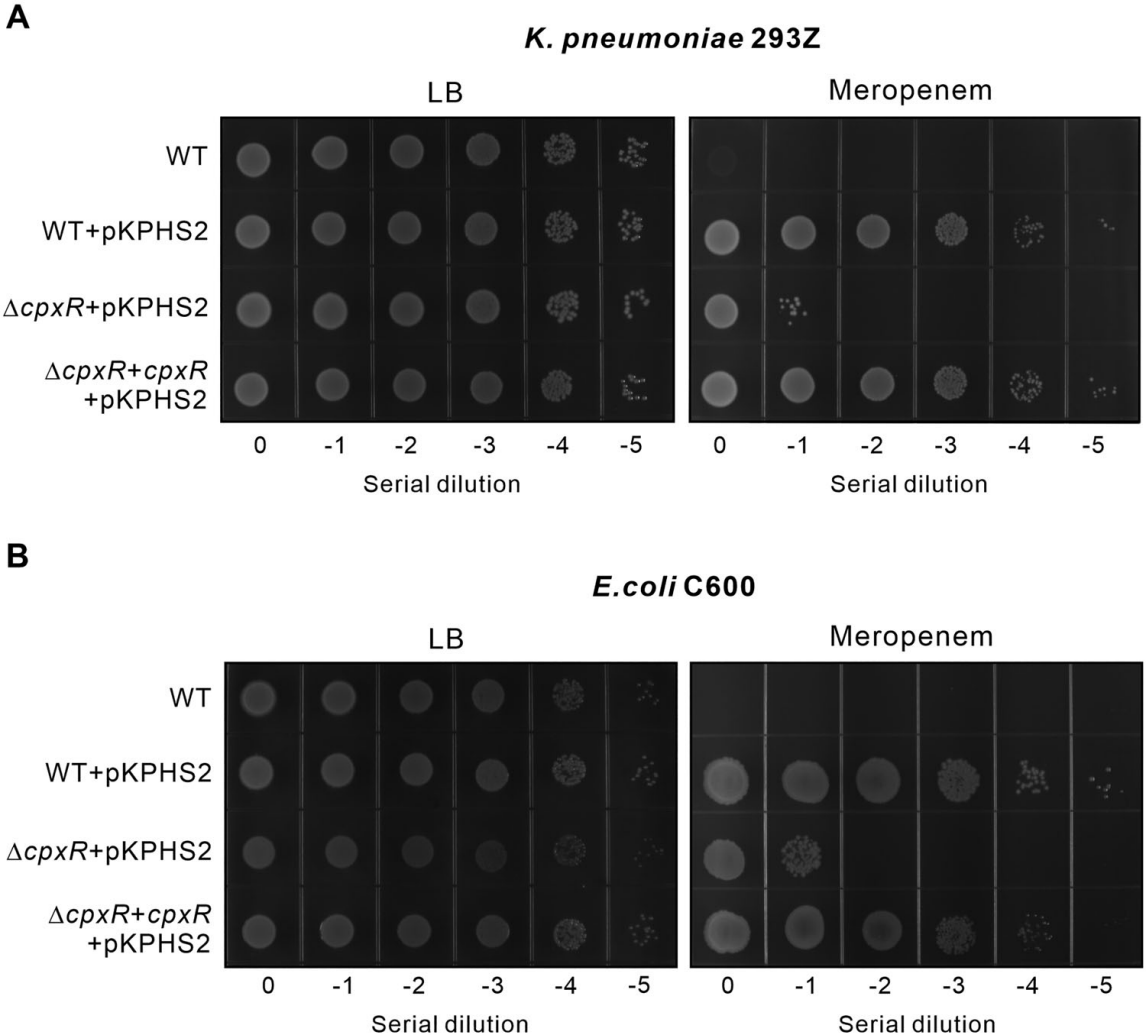
CpxR regulates meropenem resistance in recipient strains containing an acquired *bla*_KPC_ gene. **(A)** Growth of *K. pneumoniae* 293Z, 293Z + pKPHS2, 293ZΔ*cpxR + *pKPHS2, 293ZΔ*cpxR *+ *cpxR + *pKPHS2 in the presence or absence of meropenem. **(B)** Growth of *E. coli* C600, C600 + pKPHS2, C600Δ*cpxR + *pKPHS2, C600Δ*cpxR *+ *cpxR + *pKPHS2 in the presence or absence of meropenem.

The transfer of *bla*_KPC_-carrying plasmids is a major reason for the spread of antibiotic resistance among Gram-negative bacteria, most of which encode a conserved *cpxR* gene on the chromosomes. To determine the contribution of CpxR in the recipient bacteria to meropenem resistance, we transferred the pKPHS2 plasmid into the hvKP strain 293Z and *E. coli* and analysed their resistance to meropenem (Figure 7, Table S5). Both hvKP and *E. coli* gained full resistance to meropenem after receiving the pKPHS2 plasmid. The resistance level was reduced by 4 logs when *cpxR* was deleted in the recipient strains, and it was restored by the complementation of *cpxR in trans*. Therefore, our results showed that CpxR is a conserved host factor crucial for carbapenem resistance mediated by the plasmid-borne *bla*_KPC_ gene.

## Discussion

**Figure 8.**
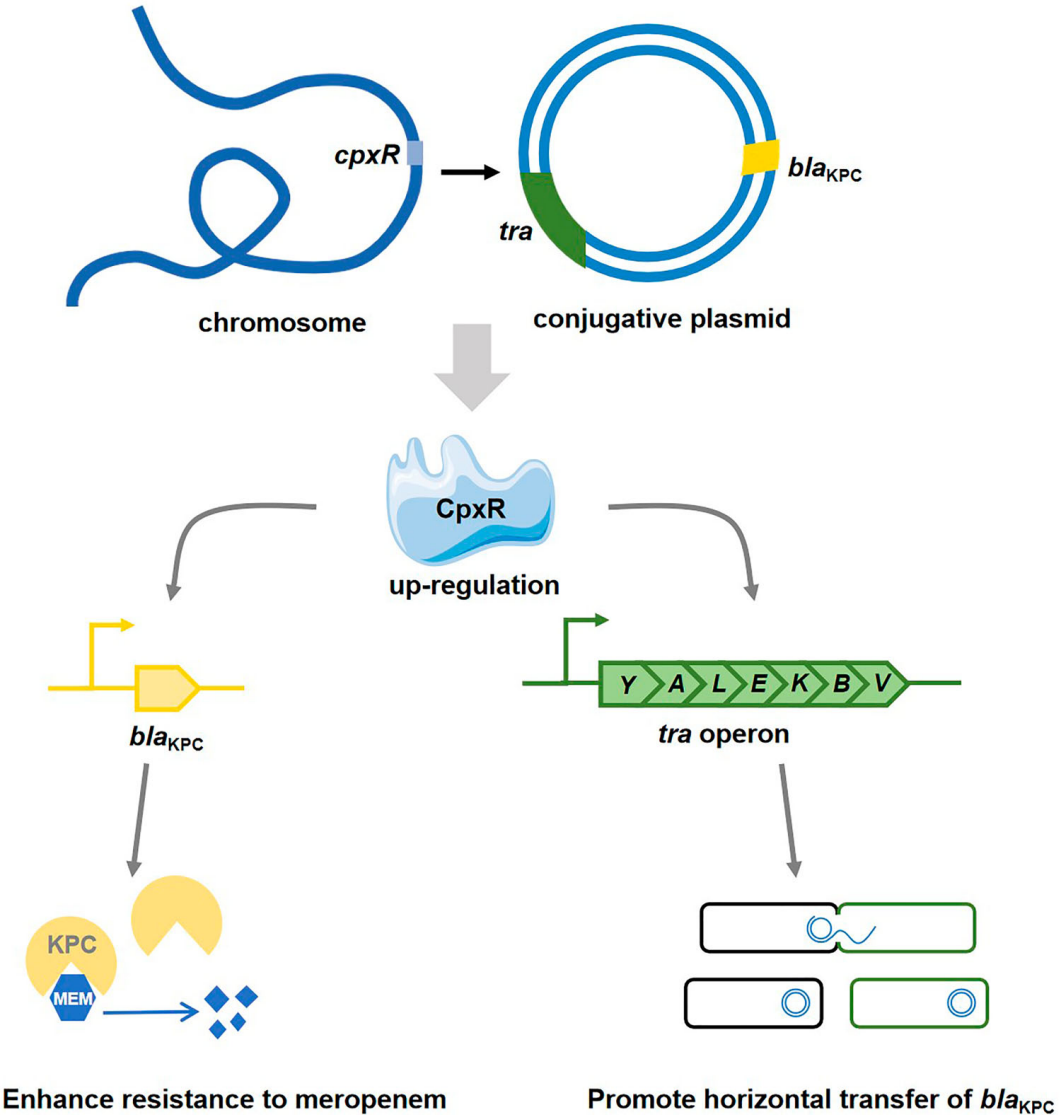
Proposed model for CpxR-mediated transcriptional regulation of *bla*_KPC_ and *tra* operon on the conjugative resistance plasmid.

Carbapenem antibiotics are one of the most important antibacterial drugs for the treatment of serious bacterial infections. Their effectiveness is compromised by the recent emergence of CRE in clinics, especially the problematic carbapenem-resistant hypervirulent *K. pneumoniae* (CR-hvKP) [33]. The carbapenem resistance is acquired commonly due to a conjugative plasmid carrying the *bla*_KPC_ gene, but the intrinsic resistance mechanism in *K. pneumoniae* has been little understood. In this study, we discovered that the chromosome-encoded TCS regulator CpxR directly regulates the expression and the mobilization of *bla*_KPC_ gene on the IncFII conjugative plasmid, playing a crucial role in the growing problem of carbapenem resistance in the classical and hypervirulent *K. pneumoniae* strains (Figure 8).

By performing an unbiased screen of twenty-four TCS knockout mutants, we discovered that CpxAR is the sole system in *Klebsiella* contributing to carbapenem resistance. CpxR, known as a key regulator of envelope stress, has been linked to the resistance of multiple classes of antibiotics in gut bacteria, including aminoglycosides [12, 34] beta-lactams [21], Fosfomycin [12] and cationic antimicrobial peptides [35]. To our knowledge, this is the first report that CpxR influences resistance to carbapenems, one of the most effective antibiotics in clinical treatments. In many previous studies, the contribution of CpxR was studied using artificially engineered variants of CpxR that are constitutively activated [18], casting some doubts on the true involvement of CpxR in antibiotic resistance. Here in this study, a clean deletion of *cpxR* gene from its native chromosomal locus exhibits a clear deficit in meropenem resistance, which was fully rescued by CpxR provided *in trans*. Our results unequivocally demonstrated that the endogenous CpxR plays an indispensable role in antibiotic resistance in bacteria.

CpxR promotes carbapenem resistance by a direct regulation of resistance genes *bla*_KPC_ in *Klebsiella.* Our data showed that CpxR binds to the promoter region of *bla*_KPC_ (Figures 2 and 3), and activates the transcription of *bla*_KPC_ in the IncFII plasmid of *K. pneumoniae* ST11 (Figure 3). It was previously reported that CpxR mainly acts on the envelope permeability to limit intracellular antibiotic concentrations, either by activating efflux pumps [21–23], or regulating major membrane porins [20, 21]. The direct regulation of *bla*_KPC_ gene provides a new mechanism for how CpxR influences resistance to antibiotics. Such direct regulations have been documented for other TCS in different bacteria. For example, the VbrKR system in *V. parahaemolyticus* was shown to activate the expression of class A β-lactamase (*blaA*^VP^, *vpa0477*) to confer ampicillin resistance [36].

Different from the chromosomally encoded *blaA*^VP^, the *bla*_KPC_ gene is located on a mobile genetic element (*i.e.* the IncFII conjugative plasmid) that may circulate in numerous enteric bacterial species. It was anticipated that the regulation of mobile *bla*_KPC_ would be conserved in different bacterial hosts in which CpxR is conserved [11]. Investigating this hypothesis, we have proven that 1) the *bla*_KPC_ can be transferred to new bacterial species via conjugation, 2) the newly acquired *bla*_KPC_ gene is regulated by the host CpxR in the recipient bacteria (Figure 7, Table S5). Strikingly, we found that CpxR facilitates the transmission of pKPHS2 between cKP, hvKP and *E. coli* (Figure 4), promoting the dissemination of *bla*_KPC_ genes via horizontal transfer. The IncFII conjugative plasmid pKPHS2 encodes an entire F-type Type IV secretion system (T4SS), which makes the *bla*_KPC_ highly susceptible to strain-to-strain transmission by conjugation. CpxR activates the expression of *tra* genes that are required for conjugative transfer. CpxR directly binds to the promoter of the *tra* operon (P*_traY_*), upregulates the transcription of *tra* genes, and facilitates the mobilization of pKPHS2 and the plasmid-encoded *bla*_KPC_ (Figures 4–6). When pKPHS2 is transferred to other Enterobacterales, CpxR acts as the key host factor promoting carbapenem resistance in recipient bacteria (Figure 7, Table S5). CpxAR is a highly conserved TCS in the core genomes in Enterobacterales. Our analysis of 1280 *K. pneumoniae* complete genome sequences currently available on GenBank confirmed the presence of intact *cpxAR* genes (Figure S14). Thus, the cross-regulation of mobile *bla*_KPC_ by host CpxR may likely represent a ubiquitous regulatory mechanism, which contributes to the ongoing emergence of the carbapenem antibiotics resistance of classical and hypervirulent *K. pneumoniae*.

CpxR regulates transcription by binding directly to the promoter region of target genes. The canonical binding sites were thought to resemble a sequence motif GTAAA-(N)_4-8-_GTAAA [11, 37–39]. However, in recent years, an increasing number of different CpxR motifs have been identified, such as GTATT-N_5_-GAAAA [35], GAAAT-N_5_-GTAAA [35], TTAAA-N_6_-GTAAA [40], TTGAC-N_5_-CTTGC [14] and TGAAA-N_3_-TTTAT [16] among others (Table S5). In this study, three CpxR binding sites were identified by DNaseI footprinting, each containing the new motifs TTAAT-N_6_-ATAAT, AAATA-N_5_-AAATA, and TTAAA-N_5_-GTAAT, respectively. These results expand our understanding of the CpxR binding sites and various genes directly regulated by CpxR.

## Summary

By screening twenty-four putative TCS encoded on the chromosome of *K. pneumoniae* HS11286, we have discovered that the CpxAR TCS is required in the carbapenem resistance. CpxR not only upregulated the expression of the plasmid-carrying gene *bla*_KPC_ but also promoted the conjugation of *bla*_KPC_-carrying plasmid pKPHS2 between the cKP, hvKP and *E. coli* strains. As a highly conserved regulatory system in many bacteria, the CpxR may contribute to the transmission of carbapenem antibiotics resistance among Enterobacteriaceae.

## Supplementary Material

Supplemental Material

Supplemental Material

Supplemental Material

Supplemental Material

Supplemental Material

Supplemental Material

Supplemental Material
