# Supplementary material for: CpxR promotes the carbapenem antibiotic resistance of Klebsiella pneumoniae by directly regulating the expression and the dissemination of blaKPC on the IncFII conjugative plasmid
Source: Emerg Microbes Infect. 2023 Sep 6;12(2):2256427. doi: 10.1080/22221751.2023.2256427 (PMC10524804; doi:10.1080/22221751.2023.2256427)
Supplement: Supplemental Material [file TEMI_A_2256427_SM2314.docx]

**CpxR promotes the carbapenem antibiotic resistance of *Klebsiella pneumoniae* by directly regulating the expression and the dissemination of *bla*_KPC_ on the IncFII conjugative plasmid**

**SUPPLEMENTARY METHODS**

Construction of gene knockout strains

Construction of pXG10-based complementation plasmids

Construction of pET28a(+)-based mutants protein expression plasmids

**SUPPLEMENTARY DATA**

Table S1. Strains and plasmids used in this study

Table S2. Primes used in this study

Table S3. Antibiotics and the corresponding concentrations used in conjugation assay.

Table S4. P2CS-predicted two-component systems in *K. pneumoniae* HS11286.

Table S5. Minimal inhibitory concentrations (MICs) of the wild-type, *cpxR* mutant and *cpxR* complement strains.

Table S6 CpxR motifs.

Figure S1. Polymerase chain reaction (PCR) to confirm the knockout of 24 TCS genes.

Figure S2. *cpxAR* is a bicistronic operon on the chromosome of *K. pneumoniae* HS11286.

Figure S3. Genetic organization of wild-type CpxR and mutants in *K. pneumoniae* HS11286.

Figure S4. Workflow for *in silico* analysis of the CpxR binding sequences upstream of *bla*_KPC_ and other β-lactamase genes in *K. pneumoniae* plasmids.

Figure S5. Sequence analysis of the *bla*_KPC_ gene promoter regions in *K. pneumoniae* plasmids.

Figure S6. The relationship between the *bla*_KPC_ (or *bla*_TEM_) gene promoter regions with CpxR binding sites, plasmid incompatibility group, and strain sequence typing of *K. pneumoniae.*

Figure S7. Electrophoretic mobility shift assay of CpxR with 2 pmol FAM labeled promoter DNA of *bla*_KPC_ of Tn*4401*b in the carbapenem resistance plasmid of ST258 *K. pneumoniae.*

Figure S8. Electrophoretic mobility shift assays negative controls.

Figure S9. Western blot to detect the expression of *K. pneumoniae* carbapenemase (KPC) protein with or without *cpxR.*

Figure S10. Determining the location of the *tra* operon promoter on the *bla*_KPC_-carrying plasmid pKPHS2.

Figure S11. Electrophoretic mobility shift assay of CpxR with 2 pmol FAM labeled promoter region of *traY* and 68 pmol unlabeled promoter region of *traY* (Negative control).

Figure S12. Conjugation of *bla*_KPC_-carrying plasmid pKPHS2 between *E. coli* and *K. pneumoniae.*

Figure S13. Verification of the *bla*_KPC_-carrying plasmid (pKPHS2) conjugation between *E. coli* C600 and *K. pneumoniae* RJF293.

Figure S14. Conservation analysis of 24 two-component systems (TCSs) across the genomes of *K. pneumoniae.*

**SUPPLEMENTARY METHODS**

**Construction of gene knockout strains**

Overnight cultures of gene knockout *E. coli* or *K. pneumoniae* strains containing the pKOBEG-Apr plasmid were prepared in lysogeny broth (LB) medium supplemented with 50 µg/ml apramycin. The cultures were incubated overnight at 30°C. Subsequently, a 1:100 dilution of the overnight cultures was transferred to fresh LB broth. When the OD_600nm_ reached 0.2, a final concentration of 0.2% L-(+)-arabinose was added and incubated for 2 hours at 30°C. The bacterial cultures were then collected and processed to obtain electrocompetent cells.

To replace the target gene, the template DNA corresponding to the desired modification was amplified using a splicing overlap extension (SOE) PCR method. Specific primers (listed in Table S2) were used for this amplification step. The electrocompetent cells were transformed with the amplified template DNA using 0.2-cm electroporation chambers and the transformation parameters were set to 200 Ω, 25 µF, and 2.5 kV. Following transformation, the cells were selected on LB plates supplemented with 200 µg/ml hygromycin and incubated overnight at 37°C. Colonies that appeared on the plates were subjected to PCR analysis to confirm the successful replacement of the target gene with the hygromycin resistance gene (*hph*) and the loss of the pKOBEG-Apr plasmid.

To further eliminate the *hph* gene from the confirmed mutant strain, electrocompetent cells were prepared and transformed with the pFLP2-Apr plasmid. The transformed cells were then cultured overnight at 37°C on LB plates supplemented with 50 µg/ml apramycin. From the resulting colonies, a single apramycin-resistant colony was selected and streaked onto LB plates containing 6% sucrose. The plates were incubated at 37°C to promote the curing of the pFLP2-Apr plasmid. The colonies obtained from this step were verified through patching and PCR to confirm the absence of the *hph* gene.

**Construction of pXG10-based complementation plasmids**

The linear vector pXG10 was amplified by using the primers pXG10-Apr-FU-F/R (listed in Table S2). Then the PCR products were digested with DpnI enzyme.

Using *K. pneumoniae* HS11286 genomic DNA (gDNA) as a template, PCR was performed with primers cpxR-101-FU-F/R (listed in Table S2) to amplify DNA fragments includes the promoter of *cpxR* and the *cpxR* gene.

Using *K. pneumoniae* HS11286 gDNA as a template, PCR was performed with primers cpxA-101-FU-F/R (listed in Table S2) to amplify DNA fragments includes the promoter of *cpxA* and the *cpxA* gene.

Using *K. pneumoniae* HS11286 gDNA as a template, PCR was performed with primers DEC-101-FU-F/R (listed in Table S2) to amplify DNA fragments includes the promoter of *cpxR* and the *cpxR*^NTD^ gene (DNA fragments corresponding to amino acids 1^st^-131^th^ of CpxR).

Using *K. pneumoniae* HS11286 gDNA as a template, PCR was performed respectively using primers cpxR-101-FU-F / cpxR-R195H-R and cpxR-101-FU-R / cpxR-R195H-F (listed in Table S2) to amplify two DNA fragments containing the desired point mutation. Subsequently, the splicing overlap extension (SOE) PCR was used to connect the two DNA fragments, resulting DNA fragments includes the promoter of *cpxR* and the *cpxR*^R195H^ gene (Arg195 residue mutation to a histidine).

All the obtained DNA fragments possessed 15-base pair sequences at both ends, which are identical to the corresponding sequences found at the ends of the linear vector pXG10.Subsequently, the linear vector pXG10 and the aforementioned DNA fragments were ligated together using Gibson Assembly^®^ (NEB) through seamless cloning, resulting in the construction of plasmids pXG10-Apr-*cpxR*, pXG10-Apr-*cpxA*, pXG10-Apr-*cpxR*^NTD^ and pXG10-Apr-*cpxR*^R195H^.

**Construction of pET28a(+)-based mutants protein expression plasmids**

1. CpxR NTD: pET28a(+) plasmid was digested with NdeI and HindIII enzymes to linearize the vector. Using *K. pneumoniae* HS11286 gDNA as a template, PCR was performed with primers cpxR-Re-FU-F/R (listed in Table S2) to amplify DNA fragments corresponding to amino acids 1^st^-131^th^ of CpxR. The resulting DNA fragment had approximately 15-base pair sequences at both ends, which were identical to the corresponding sequences at the ends of the linear vector pET28a(+). Subsequently, the linear vector pET28a(+) and the DNA fragments corresponding to amino acids 1^st^-131^th^ of CpxR were ligated together using Gibson Assembly® (NEB) for seamless cloning.
2. CpxR R195H: A splicing overlap extension (SOE) PCR was employed to create this mutation. Using *K. pneumoniae* HS11286 gDNA as a template, PCR was performed respectively using primers cpxR-pET28-FU-F / cpxR-R195H-R and cpxR-pET28-FU-R / cpxR-R195H-F (listed in Table S2) to amplify two DNA fragments containing the desired point mutation. Subsequently, the SOE PCR was used to connect the two DNA fragments, resulting in *cpxR*^R195H^ DNA fragments with approximately 15-base pair sequences at both ends, which matched the corresponding sequences at the ends of the linear vector pET28a(+). Finally, the *cpxR*^R195H^ DNA fragments were cloned into the linear vector pET28a(+) using the same aforementioned method.

**SUPPLEMENTARY DATA**


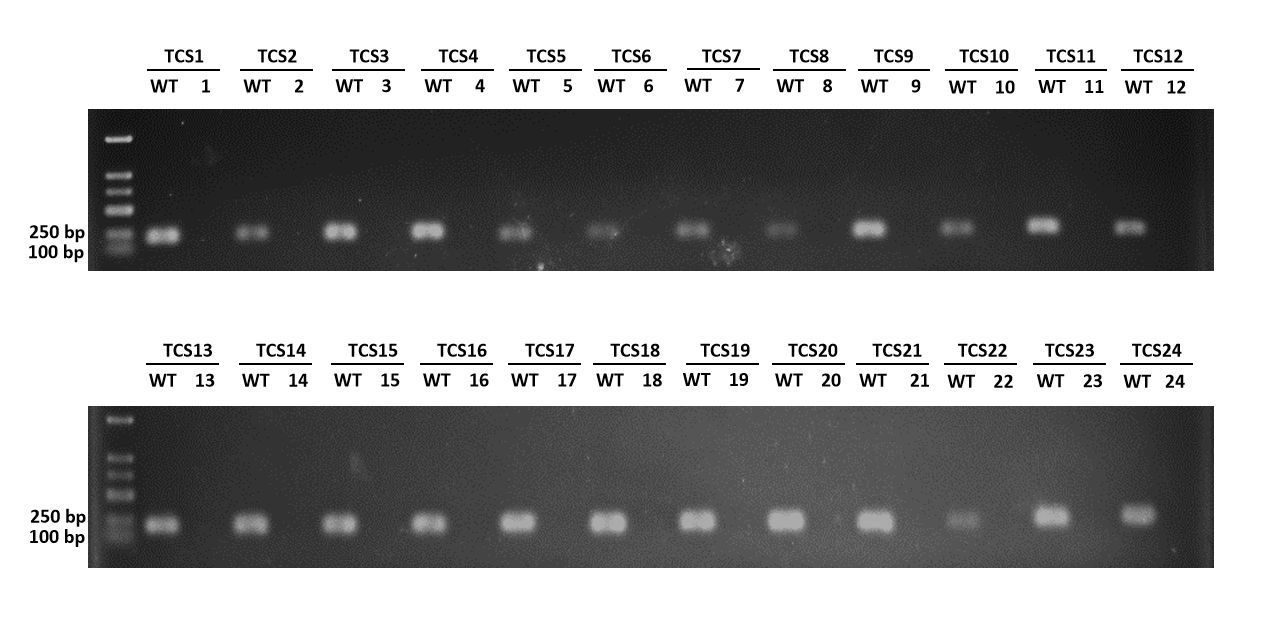
Figure S1**.** Polymerase chain reaction (PCR) assay to confirm the knockout of 24 TCS genes. PCR was conducted using the specific primers designed to amplify the gene deletion region. If DNA product was obtained from the wild-type but not from the mutant, it was considered successful construction of the mutant. The primers used for this were listed in Table S2, including TCS1-F/R to TCS24-F/R.


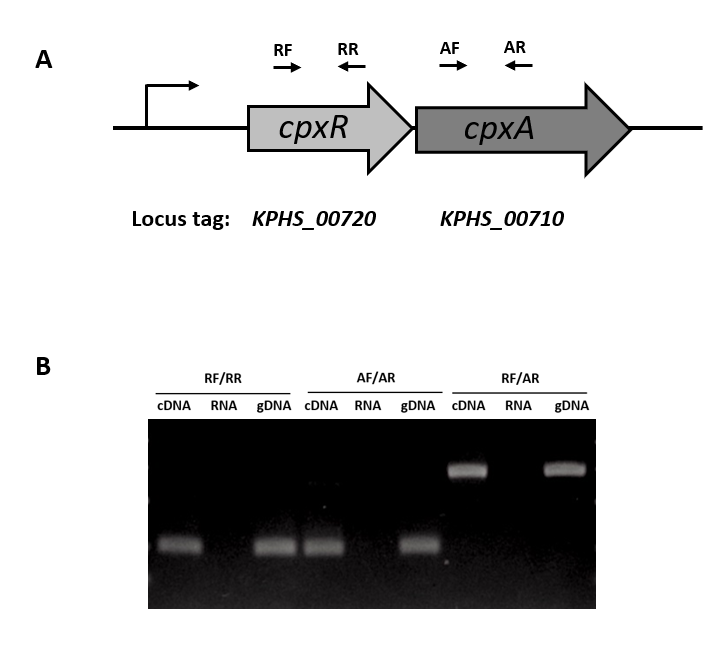


Figure S2**.** *cpxAR* is a bicistronic operon on the chromosome of *K. pneumoniae* HS11286. **(A)** Genetic organization of the *cpxAR* locus. **(B)** RT-PCR showed the co-transcription of the *cpxAR* locus. Primers were shown in (A) and were listed in Table S2.


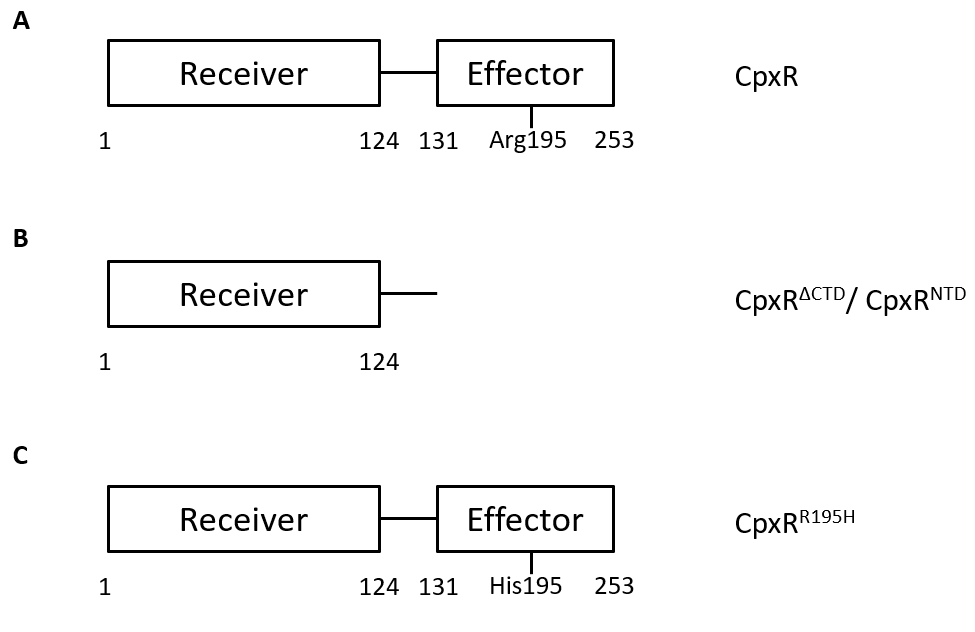
Figure S3. Genetic organization of wild-type CpxR and mutants in *K. pneumoniae* HS11286. (A) Wild-type CpxR. (B) CpxR missing the C-terminal domain. (C) Arginine 195 of CpxR was replaced by a histidine.


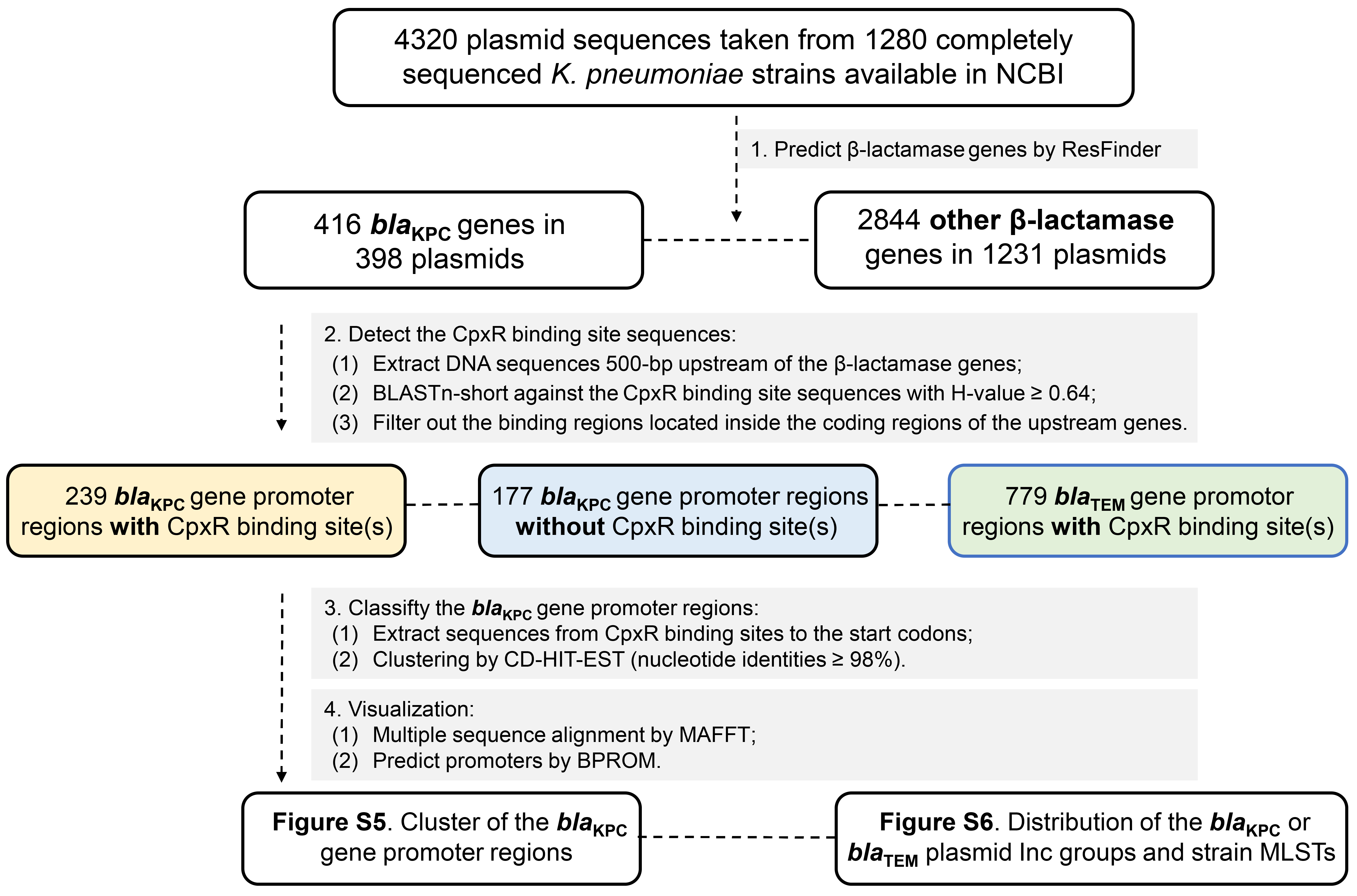


**Figure S4.** Workflow for *in silico* analysis of the CpxR binding sequences upstream of *bla*_KPC_ and other β-lactamase genes in *K. pneumoniae* plasmids (1-7). The CpxR binding site sequences were identified in the *bla*_KPC_-carrying IncFII plasmids in *K. pneumoniae* ST11 in this study, *i.e.*, 5’-TGACATATAGGTTAATGTCAT-3’ and 5’-TGTTTATTTTTCTAAATACATTCAAATATGTATC-3’. To examine the degree of sequence similarities at nucleotide level between the promoter DNA and the CpxR binding sites, we employ the NCBI BLASTn-derived *H*-value. For two nucleotide sequences under alignment, the *H*-value was calculated as follows:

$H=i\times\frac{lm}{lq}$ (1)

where *i* was the level of BLASTn identities of the region with the highest Bit score expressed as a frequency of between 0 and 1, *l_m_* the length of the highest scoring matching sequence (including gaps) and *l_q_* the query length. If there were no matching sequences with a BLASTn E value < 0.01, the *H*-value assigned to that query sequence was defined as zero.


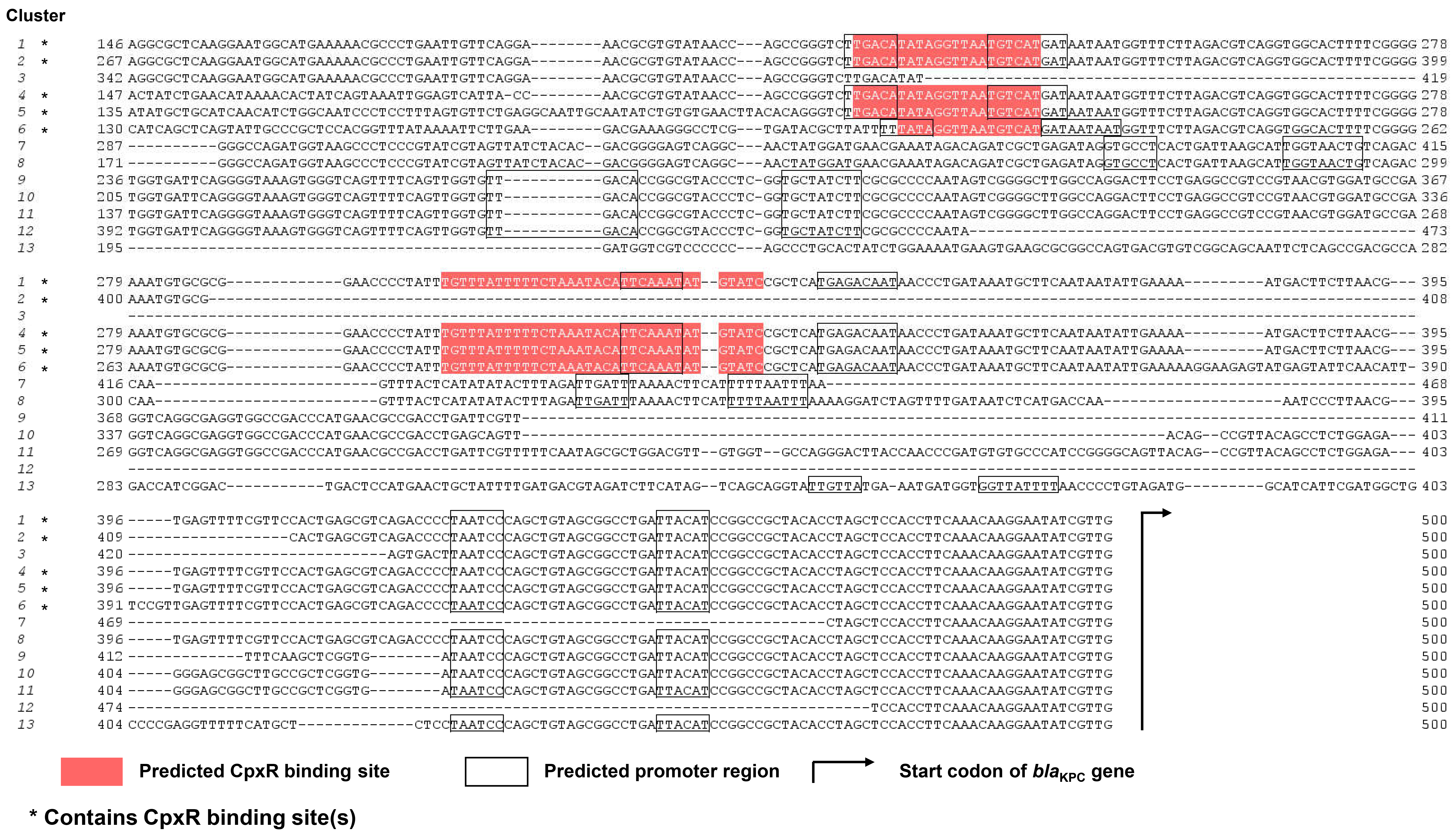


**Figure S5.** Sequence analysis of the *bla*_KPC_ gene promoter regions in *K. pneumoniae* plasmids. The upstream DNA sequences of the 416 *bla*_KPC_ genes in 398 *K. pneumoniae* plasmids were extracted and grouped by CD-HIT-EST (nucleotide identities ≥ 98%), resulting in 13 clusters. Clusters 1, 2, 4, 5 and 6 (marked with a star) have the CpxR binding sites in the *bla*_KPC_ gene promoter regions (*i.e.*, 5’-TGACATATAGGTTAATGTCAT-3’ and 5’-TGTTTATTTTTCTAAATACATTCAAATATGTATC-3’), which are identified in the *bla*_KPC_-carrying IncFII plasmids in *K. pneumoniae* ST11 in this study.


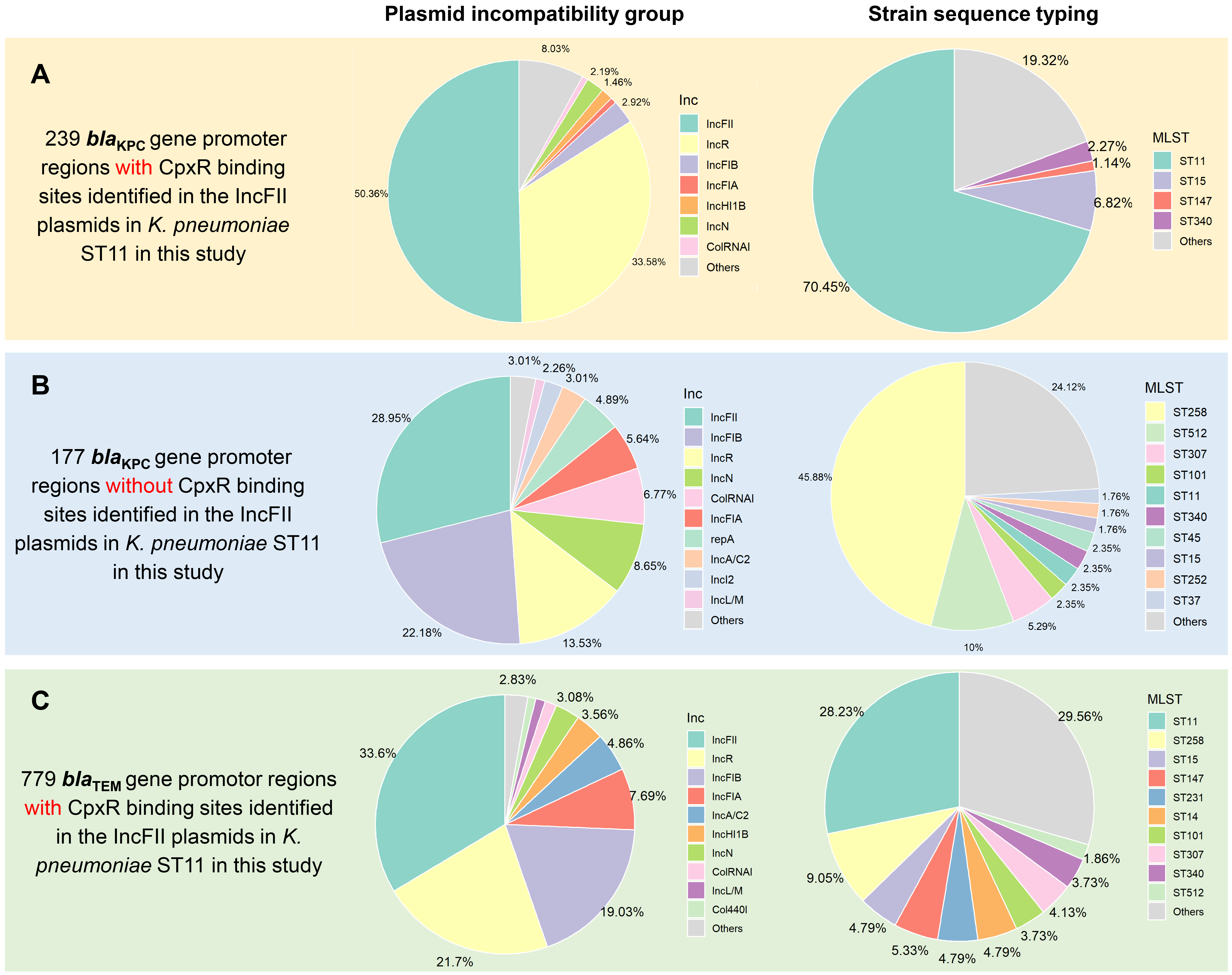


**Figure S6.** The relationship between the *bla*_KPC_ (or *bla*_TEM_) gene promoter regions with CpxR binding sites, plasmid incompatibility group, and strain sequence typing of *K. pneumoniae.* For the plasmid containing two replicon regions, the plasmid number of each Inc group was counted separately. Only the top 10 MLSTs and Inc groups with the highest frequency were displayed. The plasmid incompatibility group (Inc) and the strain sequence typing (MLST) were predicted by VRprofile2 (<https://tool2-mml.sjtu.edu.cn/VRprofile/>) (8). R package ggpubr (<https://rpkgs.datanovia.com/ggpubr/>) was utilized to generate pie charts.


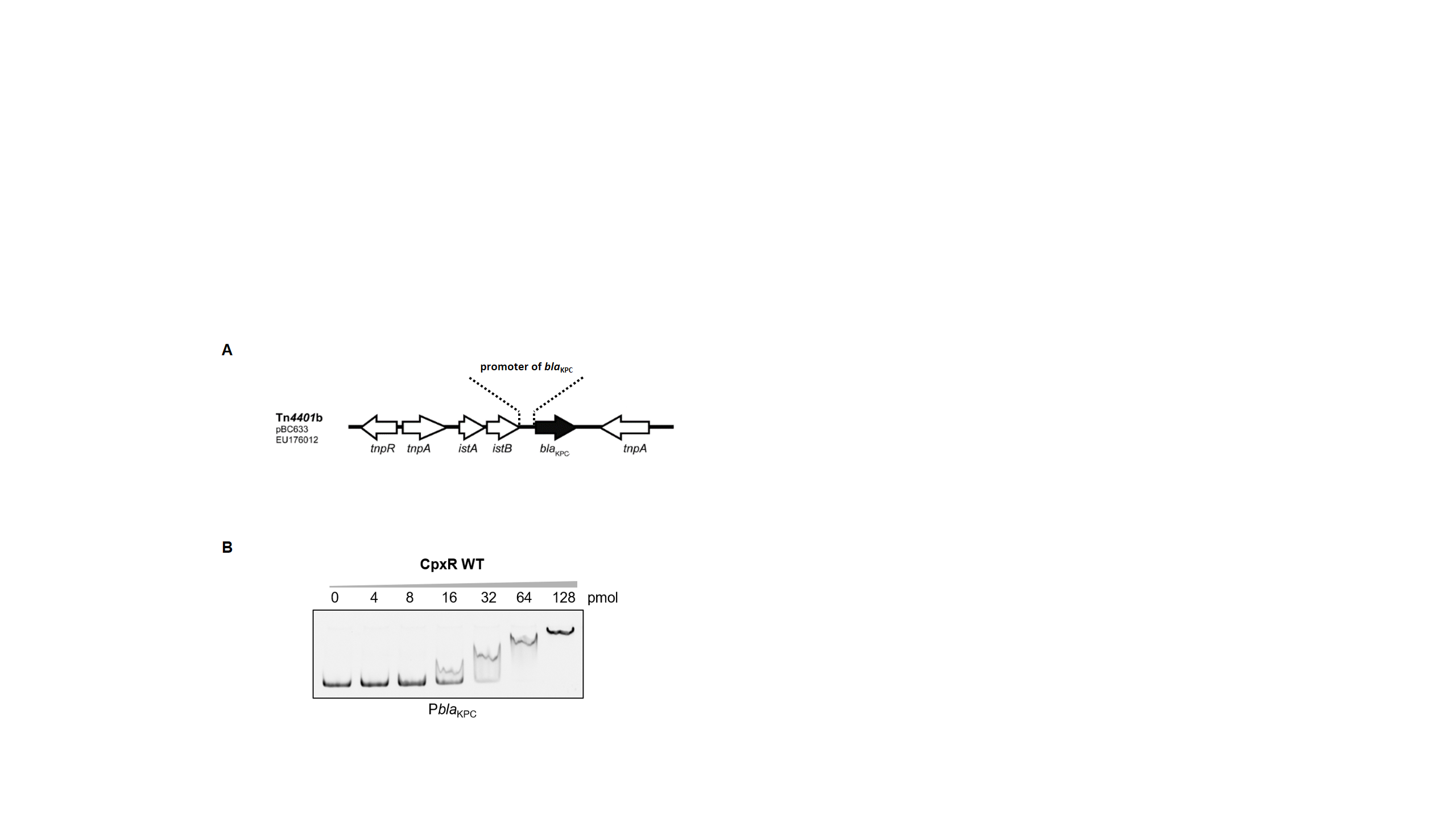
Figure S7**.** Electrophoretic mobility shift assay of CpxR with 2 pmol FAM labeled promoter DNA of *bla*_KPC_ of Tn*4401*b in the carbapenem resistance plasmid of ST258 *K. pneumoniae.*

1. The genetic organization of Tn*4401*b transposon. (B) The binding between the CpxR protein and the DNA fragment of the *bla*_KPC_ gene promoter region carried by Tn*4401*b.


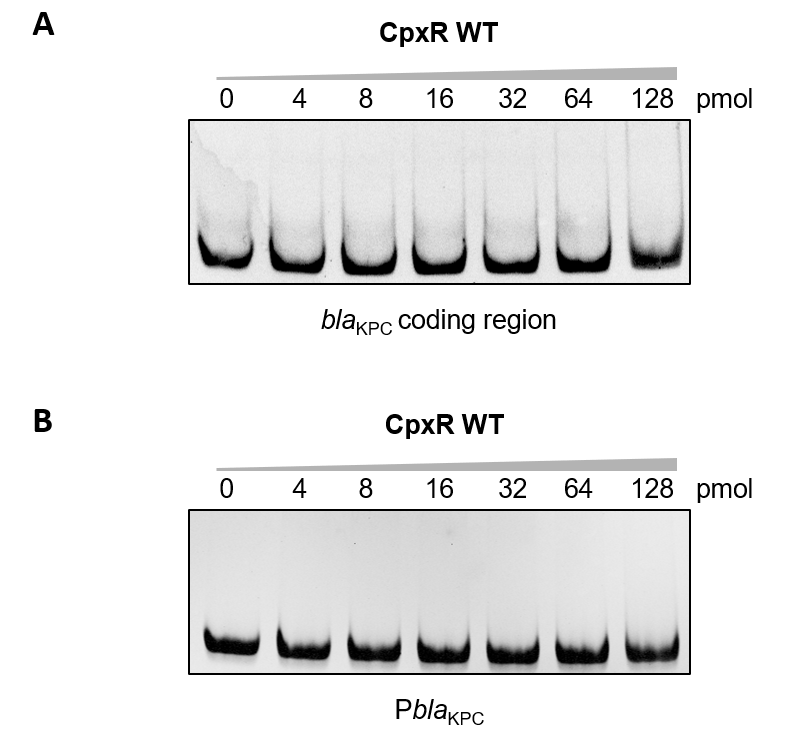
Figure S8**.** Electrophoretic mobility shift assays negative controls. **(A)** Electrophoretic mobility shift assay of increasing amounts CpxR with 2 pmol FAM labeled *bla*_KPC_ coding region. **(B)** Electrophoretic mobility shift assay of increasing amounts CpxR with 2 pmol FAM labeled promoter region of *bla*_KPC_ and 68 pmol unlabeled promoter region of *bla*_KPC_.


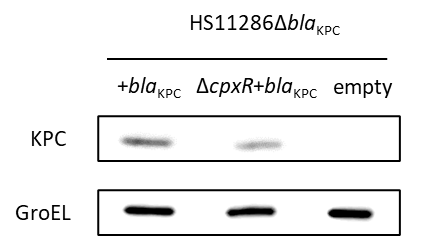


Figure S9**.** Western blot to detect the expression of *K. pneumoniae* carbapenemase (KPC) protein with or without *cpxR.* The first column denotes HS11286Δ*bla*_KPC_+*bla*_KPC_, the second column denotes HS11286Δ*bla*_KPC_Δ*cpxR*+*bla*_KPC_ and the last column denotes the negative control HS11286Δ*bla*_KPC_. The *bla*_KPC_ gene, with its promoter in N-terminal and a 3×FLAG tag in C-terminal, was cloned into the pXG10 plasmid and subsequently transformed into the *bla*_KPC_ single-deletion mutant strain HS11286Δ*bla*_KPC_, as well as the *bla*_KPC_ and *cpxR* double-deletion mutant strain HS11286Δ*bla*_KPC_Δ*cpxR*. The expression of KPC was decreased by approximately half when *cpxR* was deleted (fold change is 0.51). Equal amounts of stationary-phase bacterial cells were used for western blot analysis. KPC was detected with the Anti-Flag tag mouse monoclonal antibody (Sigma). GroEL served as a loading control in the western blot and was detected by using the Anti-GroEL antibody (Sigma) (9).


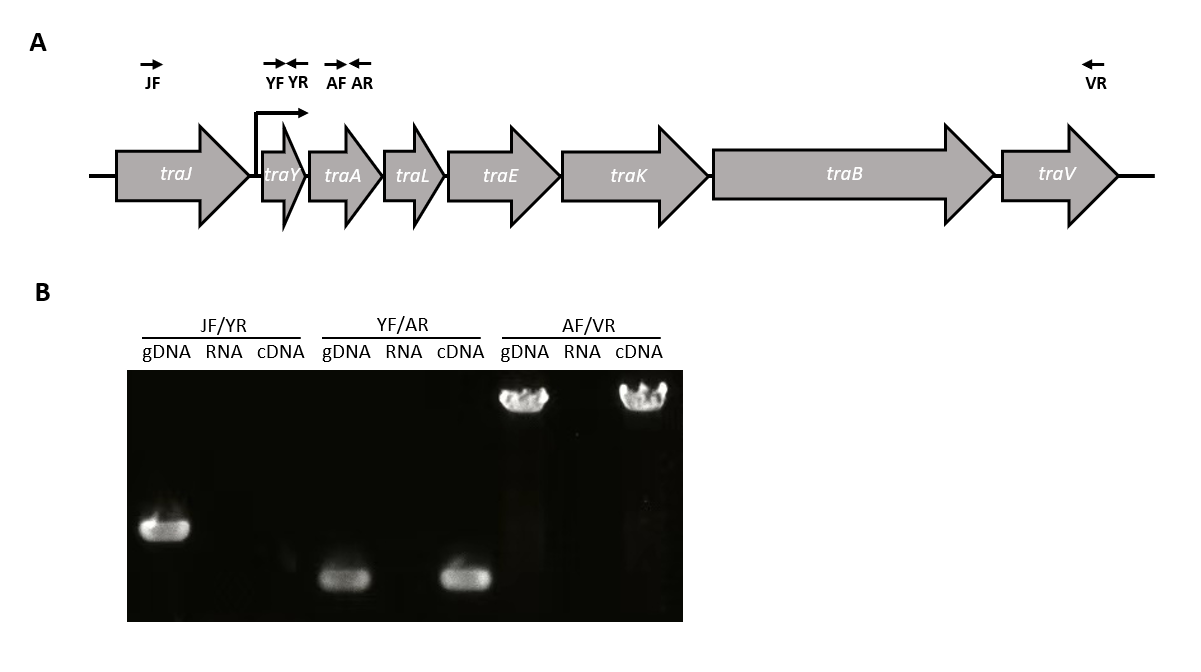
Figure S10**.** Determining the location of the *tra* operon promoter on the *bla*_KPC_-carrying plasmid pKPHS2. (A) Genetic organization of the *tra* operon. (B) RT-PCR showed that *traY*, *traA*, *traL*, *traE*, *traK,* *traB* and *traV* form a polycistron. Primers were shown in (A) and were listed in Table S2.

Figure S11**.** Electrophoretic mobility shift assay of CpxR with 2 pmol FAM labeled promoter region of *traY* and 68 pmol unlabeled promoter region of *traY* (Negative control).
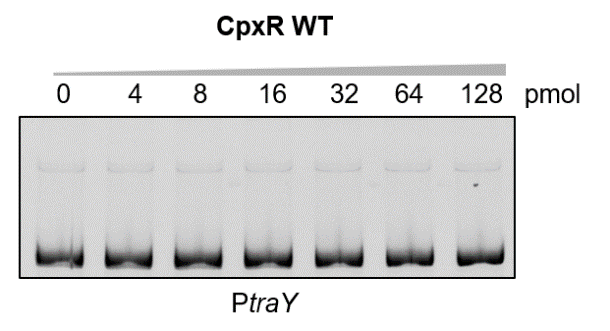


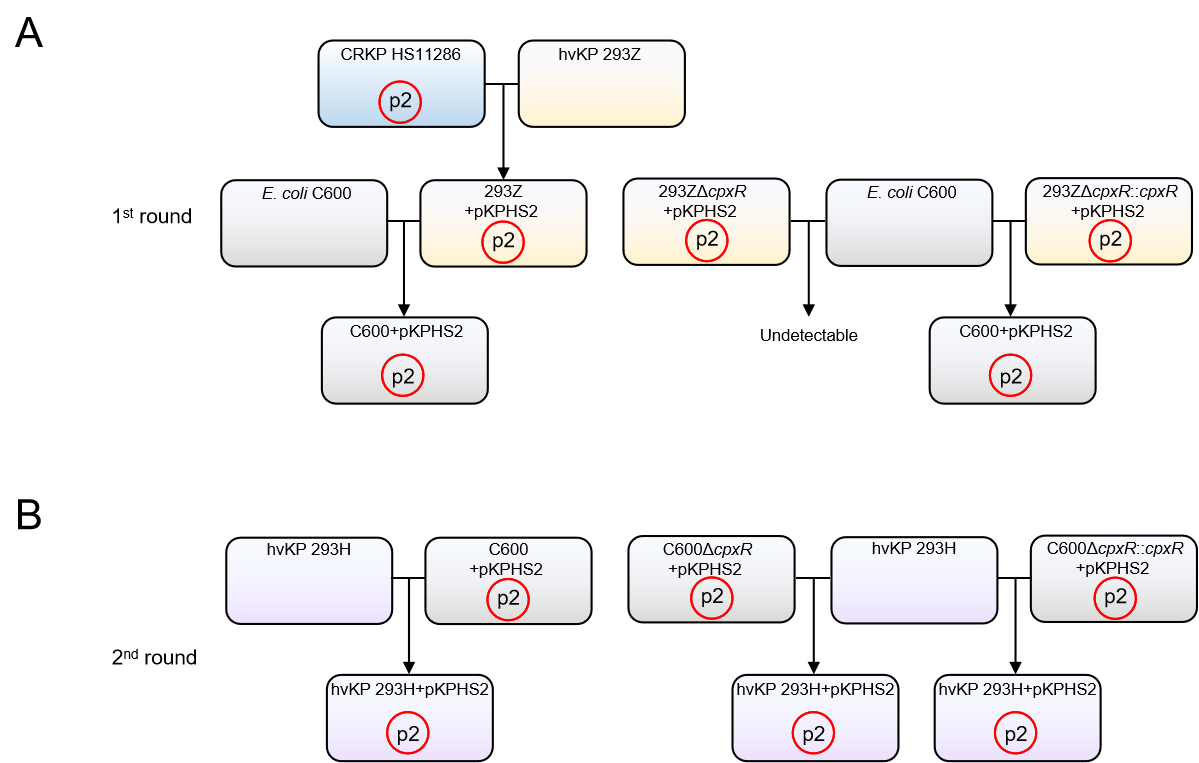
Figure S12. Conjugation of *bla*_KPC_-carrying plasmid pKPHS2 between *E. coli* and *K. pneumoniae.*


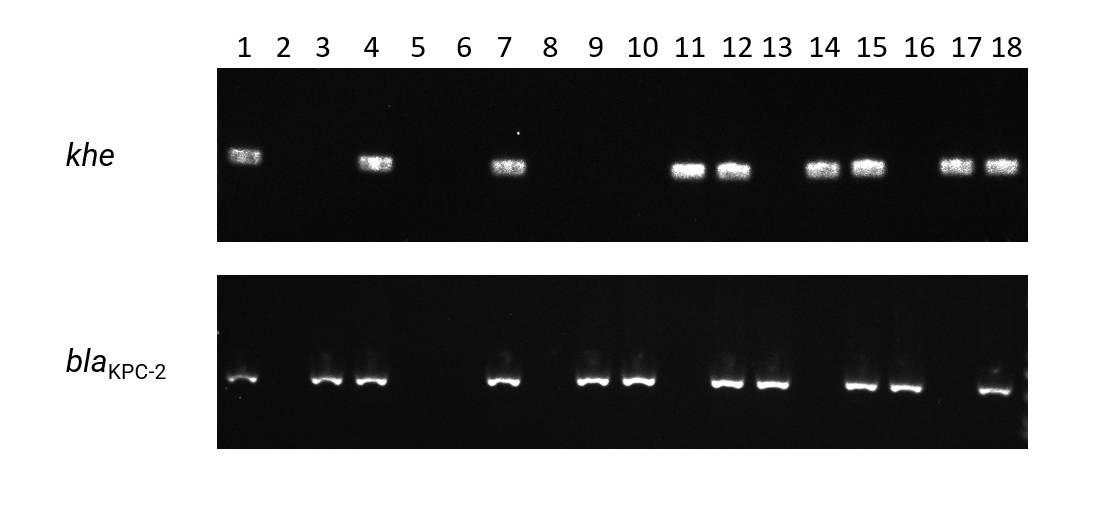


Figure S13**.** Verification of the *bla*_KPC_-carrying plasmid (pKPHS2) conjugation between *E. coli* C600 and *K. pneumoniae* RJF293. Lane 1: RJF293Z+pKPHS2, Lane 2: C600, Lane 3: Transconjugant of RJF293Z+pKPHS2 and C600; Lane 4: RJF293ZΔ*cpxR+*pKPHS2, Lane 5: C600, Lane 6: ND; Lane 7: RJF293ZΔ*cpxR*+*cpxR*+pKPHS2, Lane 8: C600, Lane 9: Transconjugant of RJF293ZΔ*cpxR*+*cpxR*+pKPHS2 and C600; Lane 10: C600+pKPHS2, Lane 11: RJF293H, Lane 12: Transconjugant of C600+pKPHS2 and RJF293H; Column13: C600Δ*cpxR*+pKPHS2, Lane 14: RJF293H, Lane 15: Transconjugant of C600Δ*cpxR+*pKPHS2 and RJF293H; Column16: C600Δ*cpxR*+*cpxR+*pKPHS2, Lane 17: RJF293H, Lane 18: Transconjugant of C600Δ*cpxR*+*cpxR+*pKPHS2 and RJF293H. The resistance gene *bla*_KPC_ located on pKPHS2 was used as a marker for the presence of plasmid. The gene *khe* exists only in *K. pneumoniae* but not in *E. coli*, which can distinguish between donor and recipient.


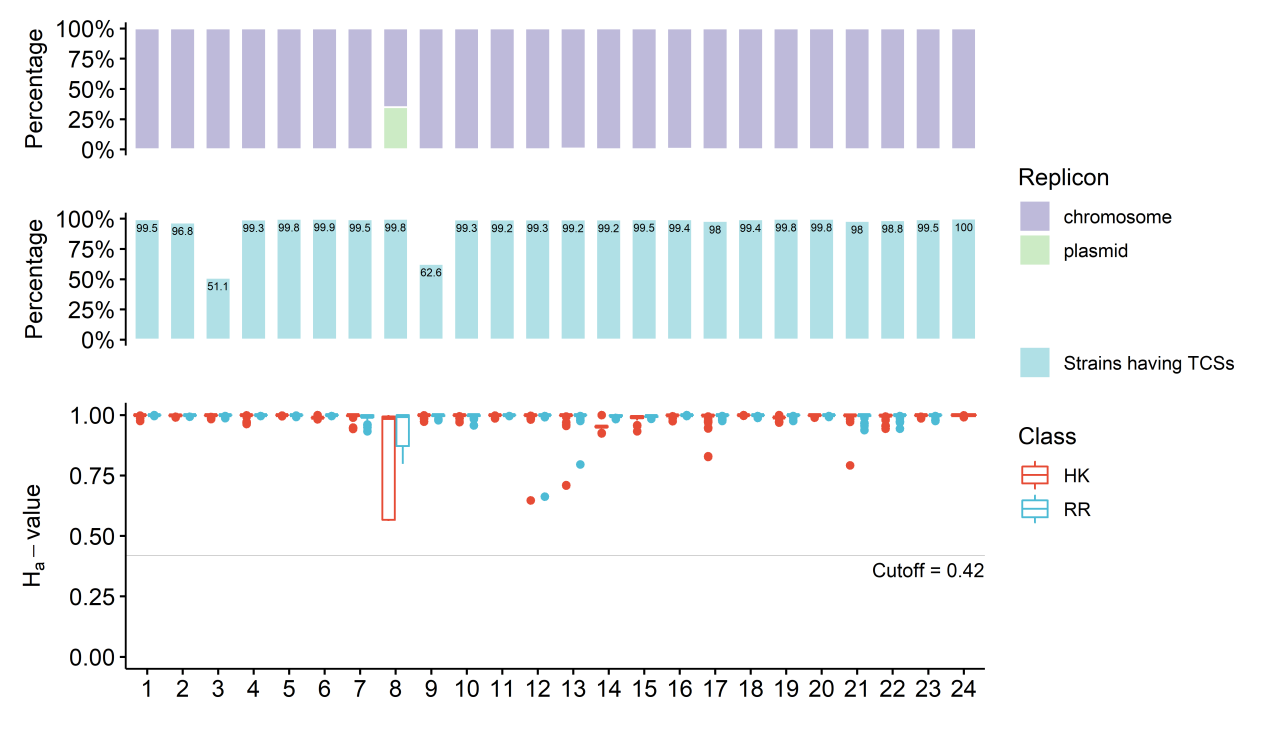


TCS

Figure S14**.** Conservation analysis of 24 two-component systems (TCSs) across the genomes of *K. pneumoniae.* These 24 TCSs are listed in Figure 1a, of which CpxAR is the sixth one. The 1280 completely sequenced genomes of *K. pneumoniae* were downloaded from GenBank in December, 2022*.* To examine the degree of sequence similarities at an amino acid level between two component proteins, we employ the NCBI BLASTp-derived *H_a_*-value. For two proteins under alignment, the *H*_a_-value was calculated as follows:

$H_{\text{a}}=i\times\frac{lm}{lq}$ (2)

where *i* was the level of BLASTp identities of the region with the highest Bit score expressed as a frequency of between 0 and 1, *l_m_* the length of the highest scoring matching sequence (including gaps) and *l_q_* the query length. If there were no matching sequences with a BLASTp E value < 0.01, the *H_a_*-value assigned to that query sequence was defined as zero. Therefore *H_a_*-value belonged to the set, *H_a_*$\in$[0,1]. In this study, a strict *H*_a_-value cut-off ≥ 0.42 was used to determine the significant sequence similarities; for example, the identities is 60% and the ratio of matching length is 70%.

**References in the supplement materials**

1. O'Leary NA, Wright MW, Brister JR, Ciufo S, Haddad D, McVeigh R, et al. Reference sequence (RefSeq) database at NCBI: current status, taxonomic expansion, and functional annotation. Nucleic Acids Res. 2016 Jan 4;44(D1):D733-D745.

2. Bortolaia V, Kaas RS, Ruppe E, Roberts MC, Schwarz S, Cattoir V, et al. ResFinder 4.0 for predictions of phenotypes from genotypes. J Antimicrob Chemother. 2020 Dec 1;75(12):3491-3500.

3. Camacho C, Coulouris G, Avagyan V, Ma N, Papadopoulos J, Bealer K, et al. BLAST+: architecture and applications. BMC bioinformatics. 2009 Dec 15;10:421.

4. Fu L, Niu B, Zhu Z, Wu S, Li W. CD-HIT: accelerated for clustering the next-generation sequencing data. Bioinformatics (Oxford, England). 2012 Dec 1;28(23):3150-3152.

5. Solovyev V, Salamov A. Automatic annotation of microbial genomes and metagenomic sequences. Nova Science Publishers, 2011: 62-78.

6. Rozewicki J, Li S, Amada KM, Standley DM, Katoh K. MAFFT-DASH: integrated protein sequence and structural alignment. Nucleic Acids Res. 2019 Jul 2;47(W1):W5-W10.

7. Waterhouse AM, Procter JB, Martin DM, Clamp M, Barton GJ. Jalview Version 2--a multiple sequence alignment editor and analysis workbench. Bioinformatics (Oxford, England). 2009 May 1;25(9):1189-1191.

8. Wang M, Goh Y, Tai C, Wang H, Deng Z, Ou H, et al. VRprofile2: detection of antibiotic resistance-associated mobilome in bacterial pathogens. Nucleic Acids Research. 2022 Jul 5;50(W1):W768-W773.

9. Chao Y, Vogel JR. A 3' UTR-Derived Small RNA Provides the Regulatory Noncoding Arm of the Inner Membrane Stress Response. Molecular Cell. 2016 Feb 4;61(3):352-363.
